# Aging induces cardiac mesenchymal stromal cell senescence and promotes endothelial cell fate of the CD90 + subset

**DOI:** 10.1111/acel.13015

**Published:** 2019-07-29

**Authors:** Hélène Martini, Jason S. Iacovoni, Damien Maggiorani, Marianne Dutaur, Dimitri J. Marsal, Jerome Roncalli, Romain Itier, Camille Dambrin, Nathalie Pizzinat, Jeanne Mialet‐Perez, Daniel Cussac, Angelo Parini, Lise Lefevre, Victorine Douin‐Echinard

**Affiliations:** ^1^ Inserm UMR Institute of Cardiovascular and Metabolic Diseases Toulouse France; ^2^ Institute Cardiomet, FHU IMPACT University Hospital of Toulouse Toulouse France; ^3^ Paul Sabatier University Toulouse France

**Keywords:** CD90, heart, IL‐1ß, macrophages, mesenchymal stromal cells, senescence

## Abstract

Aging is a major risk factor in the development of chronic diseases, especially cardiovascular diseases. Age‐related organ dysfunction is strongly associated with the accumulation of senescent cells. Cardiac mesenchymal stromal cells (cMSCs), deemed part of the microenvironment, modulate cardiac homeostasis through their vascular differentiation potential and paracrine activity. Transcriptomic analysis of cMSCs identified age‐dependent biological pathways regulating immune responses and angiogenesis. Aged cMSCs displayed a senescence program characterized by *Cdkn2a* expression, decreased proliferation and clonogenicity, and acquisition of a senescence‐associated secretory phenotype (SASP). Increased CCR2‐dependent monocyte recruitment by aged cMSCs was associated with increased IL‐1ß production by inflammatory macrophages in the aging heart. In turn, IL‐1ß induced senescence in cMSCs and mimicked age‐related phenotypic changes such as decreased CD90 expression. The CD90+ and CD90‐ cMSC subsets had biased vascular differentiation potentials, and CD90+ cMSCs were more prone to acquire markers of the endothelial lineage with aging. These features were related to the emergence of a new cMSC subset in the aging heart, expressing CD31 and endothelial genes. These results demonstrate that cMSC senescence and SASP production are supported by the installation of an inflammatory amplification loop, which could sustain cMSC senescence and interfere with their vascular differentiation potentials.

## 1 INTRODUCTION

Aging represents a dominant risk factor for developing chronic diseases, especially cardiovascular diseases. In recent years, several proofs of concept have demonstrated that the development of age‐related diseases relies on the induction of cellular senescence. Cellular senescence is characterized by resistance to apoptosis, the expression of cell cycle inhibitors, and acquisition of a senescence‐associated secretory phenotype (SASP). Cell cycle inhibition is mainly carried out by cyclin‐dependent kinase inhibitors (CDKIs) from the INK4 family, such as p16, and from the Cip/Kip family such as p21, which inhibit S‐phase entry and cell cycle progression (He & Sharpless, 2017). In the context of cardiac aging, elimination of p16 senescent cells using INK‐ATTAC mice or senolytic treatments prevented age‐related cardiac remodeling and SASP factors production (Anderson et al., 2019; Baker et al., 2016; Lewis‐McDougall et al., 2019; Walaszczyk et al., 2019).

SASP is characterized by the production of several bioactive factors such as growth factors, pro‐inflammatory cytokines, chemokines and proteases but the fine composition of the secreted factors appears to be cell‐type‐dependent (Hernandez‐Segura et al., 2017). Whereas transient SASP production during morphogenesis and wound healing allows the coordination of several stromal cellular partners to facilitate immune infiltration, angiogenesis and limitation of scar fibrosis (Demaria et al., 2014; Storer et al., 2013), the installation of a chronic pro‐inflammatory microenvironment during aging has been shown to be deleterious with loss of tissue homeostasis and progressive organ dysfunction (Baar et al., 2017; Jurk et al., 2014). This chronic inflammatory state, named inflammaging, is associated with age‐related pathologies such as type 2 diabetes, as well as chronic kidney and cardiovascular diseases (Franceschi, Garagnani, Parini, Giuliani, & Santoro, 2018).

In the adult heart, cardiac mesenchymal stromal cells (cMSCs) participate in stromal cardiac tissue renewal by their potential to differentiate into vascular smooth muscle cells (SMC) and endothelial cells (EC) and by their ability to produce a variety of paracrine factors with trophic, angiogenic, and pro/anti‐inflammatory effects. cMSCs are related to bone‐marrow mesenchymal progenitor cells (BM‐MSCs), co‐express Sca‐1/PDGFRα markers in mice (Chong et al., 2011; Noseda et al., 2015), and have been characterized in human heart (Chong et al., 2013).

We hypothesized that aging could impact cardiac microenvironment homeostasis by inducing senescent programs in cMSCs and promoting their SASP.

In the present study, by performing transcriptional expression analysis of cell‐sorted cMSCs, we show that aged cMSCs acquire a senescent program including the expression of selective cell cycle regulators from the INK4 family and SASP factors involved in the regulation of the immune response. This senescence program is associated with modification of cMSC subset diversity and functional changes in vascular differentiation potential and monocyte chemoattraction. During aging, IL‐1ß, produced by cardiac macrophages (cMPs), could mediate paracrine senescence of cMSCs and account for CD90+ cMSC rarefaction. We show that aging is associated with specific changes in cardiac stroma microenvironment and coincides with the installation of a deleterious amplification loop promoting paracrine senescence of cMSCs and modifying their endothelial differentiation potential.

## 2 RESULTS

### 2.1 Gene expression profiling of cMSCs from young and aged C57BL/6 mice revealed specific age‐associated gene expression programs

**Figure 1:**
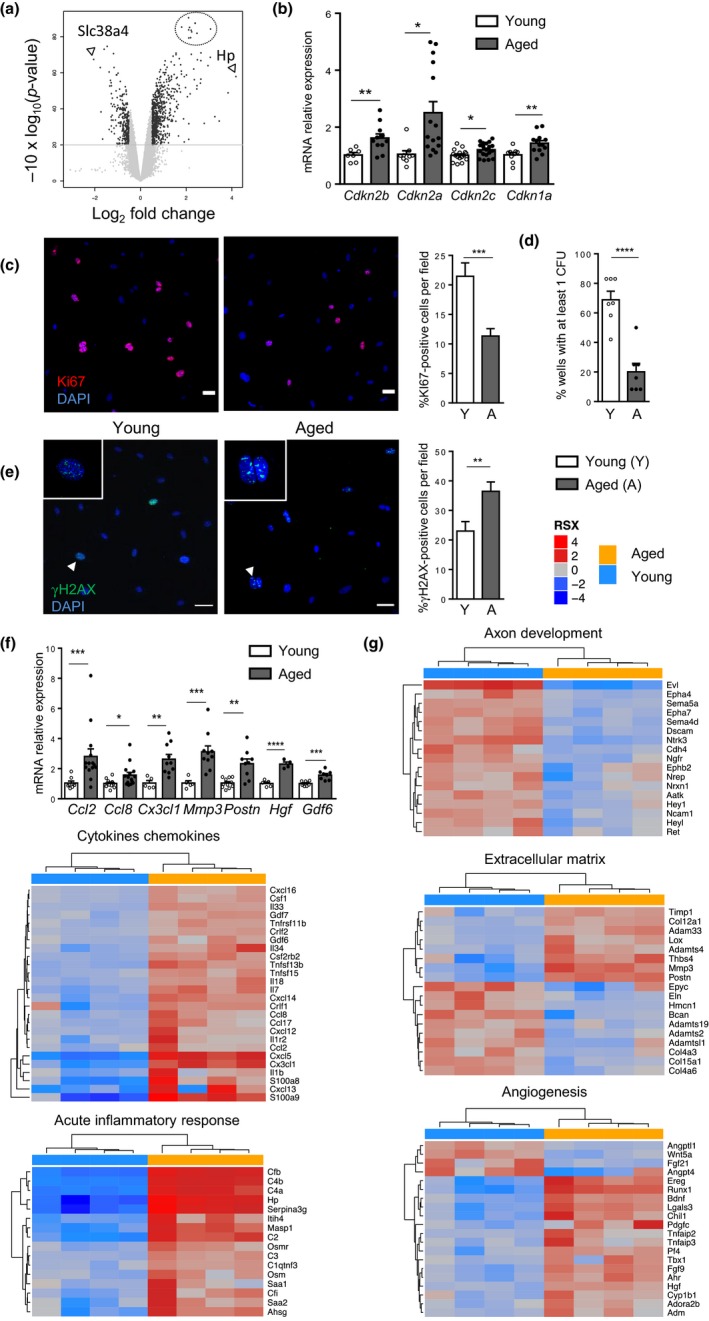
Profiling of cMSCs from young and aged C57BL/6 mice revealed specific age‐associated gene expression programs. (a) Volcano plot of differentially expressed genes between young (*n* = 4) and aged (*n* = 4) cMSCs (absolute Log_2_ FC > 0.5, *p* value < 0.01, black dots). (b) Relative mRNA expression of CDKI genes from aged cMSCs compared with young. (c) Representative immunostaining (left) with anti‐Ki67 antibody (red) and quantification (right) of proliferative young (*n* = 10) and aged (*n* = 12) cMSCs. DNA was stained with DAPI (blue). Scale bar, 50 µm. (d) Frequencies of CFU‐F from young or aged cMSCs (*n* = 7 per group). (e) Immunostaining (left) with anti‐γH2AX antibody (green) and percentage (right) of positive cells in young (*n* = 7) and aged (*n* = 8) cMSCs. DNA stained with DAPI (blue). White arrows indicated cells zoomed in. Scale bar, 50 µm. (f) Relative mRNA expression of SASP genes from aged cMSCs (*n* = 4–13) compared with young (*n* = 5–9). (g) Heatmaps of differentially expressed genes from enriched GO pathways from young (blue, *n* = 4) and aged (orange, *n* = 4) cMSCs based on microarray analysis. Data are expressed as means ± *SEM*. *p < 0.05, **p < 0.01, ***p < 0.001 versus young group

To assess the main biological pathways modified by aging in cMSCs, transcriptomic analysis by microarray was conducted on native cells after cell sorting based on the expression of Sca‐1 and CD140a (PDGFRα) and the lack of CD31 and CD45 markers (Figure S1). We identified 195 genes significantly up‐regulated and 140 genes down‐regulated in the aged group compared with young group (*p* value ≤ 0.01 and absolute log_2_ fold change > 0.5) (Figure 1a). The maximum log_2_FC value (4.1) was for haptoglobin (Hp), a protein of the acute inflammatory phase, with hemoglobin scavenger activity and immunomodulatory functions (Serrano, Luque, & Aran, 2018) (Figure 1a; Table S1). Genes with the strongest down‐regulation in aged group identified two members of the Na^+^‐dependent neutral amino acid transporter family, Slc38a4 (SNAT4) and Slc38a5 (SNAT5) (Schioth, Roshanbin, Hagglund, & Fredriksson, 2013) possibly reflecting age‐dependent changes in metabolic pathways. The best *p*‐values (<10^–7^) identified a cluster of genes up‐regulated in aged cMSCs with log_2_FC around 2 (Figure 1a; Table S1). Aged cMSCs significantly up‐regulated the expression of classical senescence‐associated CDKI genes, *Cdkn2a* (p16), *Cdkn2b* (p15), *Cdkn2c* (p18) and *Cdkn1a* (p21) (Table S1), as confirmed by RT‐PCR (Figure 1b). The higher CDKI expression in aged cMSCs was associated with lower percentage of proliferating Ki67+ cMSCs (Figure 1c) and decreased clonogenicity (Figure 1d). Aged cMSCs also accumulated DNA damage as shown by the increased percentage of γH2AX‐positive cells (Figure 1e). These results strongly supported that aged cMSCs possess an activated DNA damage response (DDR) and have triggered a senescence program with aging.

Genes down‐regulated in aged cMSCs identified gene ontology (GO) pathways involved in axonogenesis (Figure 1g) and regulation of cell death (Table 1). In contrast, up‐regulated genes in aged cMSCs defined GO pathways associated with the acquisition of SASP and regulation of the immune response (Figure 1g, Table 1). The up‐regulation of several SASP components in aged cMSCs such as chemokines (*Ccl2, Ccl8, Cx3cl1*), growth factors (*Hgf, Gdf6*), proteases (*Mmp3*) and the extracellular matrix protein periostin (*Postn*) was confirmed by RT‐PCR (Figure 1f, g).

**Table 1:** Gene Ontology (GO) pathways associated with cMSC aging

	Name	ID	*p*‐value	*q*‐value FDR B&H	Hit count
(A) GO: Biological process (Up‐regulated genes)					
1	Immune response	GO:0006955	1.92E−23	4.62E−20	95/1572
2	Cellular response to cytokine stimulus	GO:0071345	2.14E−17	1.15E−14	54/713
3	Cytokine production	GO:0001816	3.88E−15	1.87E−12	50/700
4	Secretion	GO:0046903	1.00E−11	2.09E−09	62/1228
5	Regulation of cell proliferation	GO:0042127	3.31E−11	6.38E−09	74/1666
6	Acute inflammatory response	GO:0002526	4.36E−10	6.78E−08	19/159
7	Regulation of endopeptidase activity	GO:0052548	1.52E−09	2.25E−07	29/393
8	Response to wounding	GO:0009611	5.09E−09	6.81E−07	48/967
9	Leukocyte migration	GO:0050900	6.96E−08	6.98E−06	26/386
10	Regulation of angiogenesis	GO:0045765	7.34E−08	7.15E−06	20/239
(B) GO: Biological process (Down‐regulated genes)					
1	Cell morphogenesis involved in differentiation	GO:0000904	1.04E−06	8.91E−04	25/840
2	Axonogenesis	GO:0007409	1.39E−06	9.54E−04	18/475
3	Striated muscle tissue development	GO:0014706	1.13E−05	2.28E−03	15/397
4	Positive regulation of hydrolase activity	GO:0051345	5.52E−05	5.41E−03	23/929
5	Regulation of GTPase activity	GO:0043087	6.19E−05	5.61E−03	19/688
6	Negative regulation of cell death	GO:0060548	6.22E−05	5.61E−03	24/1001

Note

Main biological processes identified by genes significantly up‐regulated (A) or down‐regulated (B) in aged cMSCs compared with young cMSCs (absolute Log_2_FC > 0.5; *p*‐value ≤ 0.01) based on microarray analysis.

Some of the biological processes defined by genes up‐regulated in aged cMSCs, such as response to wounding and angiogenesis, could be directly related to their progenitor functions. We then assessed whether aging could modify the vascular differentiation potential of cMSCs (Figure S2a‐h). Both young and aged cMSCs differentiated toward the endothelial cell lineage after culture in differentiation media but young cMSCs had higher expression of several endothelial markers at the transcriptional (*Cadh5* and *Pecam1*) and protein (vWF) levels compared with aged cMSCs (Figure S2a‐d). Conversely, SMC differentiation efficiency was higher for aged cMSCs compared with young, with higher expression of αSMA (protein and mRNA levels), *Cnn1* and *Myocd* compared with young cMSCs (Figure S2e‐h).

Together these results revealed that aging was associated with cMSCs senescence and SASP acquisition and has modified the vascular cell differentiation capability of cMSCs.

### 2.2 CCR2‐dependent chemoattraction of monocytes by aged cMSCs associated with increased frequencies of cardiac CCR2+ macrophages in aged mice

**Figure 2:**
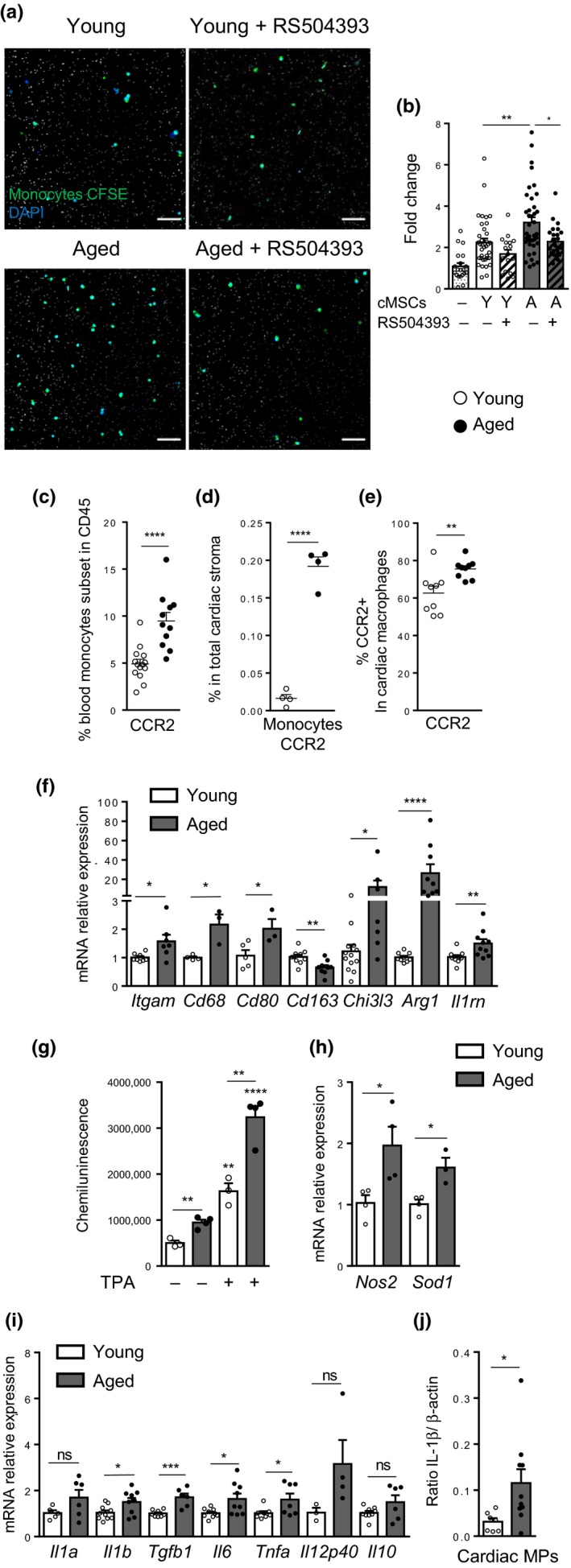
CCR2‐dependent chemoattraction of monocytes by aged cMSCs is associated with an increased frequency of cardiac CCR2+ macrophages in aged mice. (a–b) Representative imaging (a) and fold change number (b) of monocytes (CFSE: green) attracted by cMSCs from young (Y) or aged (A) mice compared with medium (pores: white; nuclei: blue). Monocytes were pretreated (or not) with the CCR2 antagonist (RS504393). Scale bar, 50 µm. (c–d) Percentage of CCR2+ monocytes in blood CD45+ cells (c) (young (*n* = 14) and aged (*n* = 11) mice) or in cardiac stromal cells (d) (*n* = 5 young and *n* = 4 aged mice). (e) Percentage of CCR2+ cells within cardiac macrophages (cMPs) (*n* = 9 young and *n* = 9 aged mice). (f–j) Expression of M1/M2 markers by young or aged cMPs. Relative mRNA expression of M1/M2 genes by young (*n* = 6–10) or aged (*n* = 6–10) cMPs (f) basal and TPA‐induced ROS production (g) and relative mRNA expression of *Nos2* and *Sod1* (h) from aged cMPs (*n* = 4) compared with young (*n* = 3–4). Relative mRNA expression of cytokines (i) genes from aged cMPs compared with young (*n* = 6–10 mice per group). Quantification of IL‐1β protein (j) in young (*n* = 7) or aged (*n* = 10) cMPs by capillary‐based Western blot normalized by β‐actin. Data are expressed as means ± *SEM*. *p < 0.05, **p < 0.01, ***p < 0.001, **** *p* < 0.0001 versus young group

The expression of chemokines (*Ccl2, Ccl8, Cxcl12*, and *Cx3cl1*) and cytokines (*Il33, Il34* and *Il7*) associated with the acquisition of a SASP by aged cMSCs could confer an increased ability of cMSCs to interact with immune cells (Figure 1g). Conversely, aged vascular endothelial cells (EC) did not up‐regulate *Ccl2* and *Ccl8* with aging (Figure S3a). As these chemokines are CCR2 ligands and known to play a major role in monocyte recruitment, we compared the ability of young and aged cMSCs to recruit monocytes using a chemotaxis assay (Figure 2a,b). Aged cMSCs attracted more monocytes than young cMSCs, and the inhibition of CCR2 by RS504393 prevented this increase (Figure 2a,b). These results showed that aged cMSCs increased monocyte recruitment through CCR2 activation, supporting a key role of these cells in cardiac monocyte recruitment.

To determine whether aging was associated with increased monocyte recruitment in the heart, blood monocytes and cardiac monocytes were analyzed by flow cytometry. Aged mice had higher percentages of circulating CCR2+ monocytes (Ly6C+ CD62L+ CX3CR1^low^) in the blood (Figure 2c, Figure S3b‐d) and of CCR2+ monocytes (Ly6C+ MHCII‐ CD64‐) in cardiac stroma compared with young mice (Figure 2d, Figure S3i). While the absolute number of cardiac macrophages (cMPs) per mice was not consistently modified with aging (Figure S3i), the frequency of CCR2+ cells in cMP population increased with age (Figure 2e; Figure S3e). The CCR2+ cMPs from aged mice had higher expression of MHCII and Ly6C compared with young (Figure S3f,g), revealing that aging favored the conversion of CCR2+ monocytes into activated CCR2+ cMPs in the heart.

To assess the potential impact of the cMP population shift on the cardiac microenvironment during aging, we cell‐sorted total cMP population and confirmed higher *Ccr2* gene expression in aged cMP pool compared with young (Figure S3j). Aging was associated with an up‐regulation in mRNA levels of both M1 (*Itgam*, *Cd68, Cd80*) and M2 markers (*Chi3l3*, *Arg1*, and *Il1rn*) (Figure 2f). Cardiac MPs from aged mice had higher production of ROS both at basal state and after NADPH oxidase stimulation (Figure 2g) which correlated with increased mRNA levels for the *Nos2* and *Sod1* enzymes (Figure 2h). Gene expression of the pro‐inflammatory cytokines, *Il1b, Tnfa* and *Il6*, and of the pro‐fibrotic cytokine *Tgfb1* also increased with aging while *Il10* was not modified (Figure 2i).

Levels of the mature form of the IL‐1ß protein also increased in cMPs with aging (Figure 2j, Figure S3k), confirming the higher propensity of aged cMPs to produce this cytokine in the cardiac microenvironment. These results show that with aging, the cMP pool is modified with increased frequencies of CCR2+ cMPs that could account for the observed M1/M2 mixed profile and the increased IL‐1ß production.

### 2.3 Treatment of cMSCs by IL‐1ß mimicked the phenotypic changes associated with aging

We hypothesized that some pro‐inflammatory mediators produced by cMPs during aging could in turn affect surrounding stromal cells and contribute to the phenotypic changes observed in aged cMSCs. Indeed, one of the GO biological processes enriched by our differential expression analysis was the “cellular response to cytokine stimulus” (Table 1, Figure S4a).

**Figure 3:**
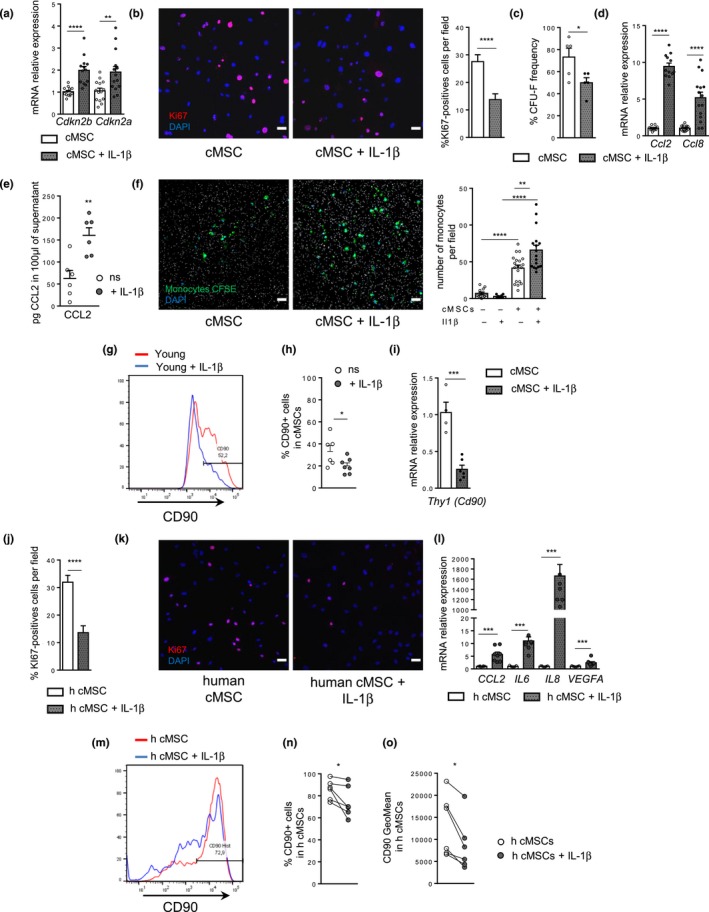
Treatment of cMSCs with IL‐1ß mimicked the phenotypic changes associated with aging. (a) Relative mRNA expression of CDKIs from cMSCs treated with IL‐1ß (+ IL‐1ß) versus untreated. (b) Representative immunostaining (left) with anti‐Ki67 antibody (red) and quantification (right) of proliferative cells in cMSCs ± IL‐1ß (Nuclei: blue). Scale bar, 50 µm. (c) Frequency of CFU‐F from cMSCs ± IL‐1ß (*n* = 5 per condition). (d) Relative mRNA expression of *Ccl2* and *Ccl8* from cMSCs + IL‐1ß versus untreated. (e) CCL2 concentration in 48h supernatants of cMSCs ± IL‐1ß (*n* = 6 per condition). (f) Representative imaging (left) and number quantification (right) of monocytes (CFSE: green) per field (pores: white, nuclei: blue). Scale bar, 50 µm. (g‐h) Representative histogram (g) and GeoMean (h) of CD90 in cMSCs treated (*n* = 7) or not (*n* = 6) with IL‐1ß. (i) Relative mRNA expression of *Thy1* (CD90) in cMSCs + IL‐1ß (*n* = 6) versus untreated (*n* = 4). (j‐o) IL‐1ß treated human cMSCs. Percentage (j) of proliferative cells and representative immunostaining (k) with anti‐Ki67 antibody (red) in human cMSCs (h cMSC) ± IL‐1ß (*n* = 3 patients). Scale bar, 50 µm. (l) Relative mRNA expression of SASP factors by h cMSC + IL‐1ß versus untreated (*n* = 3 patients). Histogram (m) and quantification (percentage (n), GeoMean (o)) of CD90 in h cMSCs ± IL‐1ß (*n* = 6 patients). Results are expressed as means ± *SEM*. *p < 0.05, **p < 0.01, ***p < 0.001, ****p < 0.0001 versus untreated group

As IL‐1R1 expression was up‐regulated in aged cMSCs at both the mRNA (Table S1) and protein levels (Figure S4b), IL‐1β could be one of the cytokines that modify gene expression of aged cMSCs. Repeated in vitro treatments of cMSCs with IL‐1ß induced the expression of *Cdkn2b* (p15) and *Cdkn2a* (p16) (Figure 3a) and decreased their proliferative and clonogenic potentials (Figure 3b,c). This treatment induced *Ccl8* and *Ccl2* gene expression (Figure 3d), secretion of CCL2 (Figure 3e) and, consequently, increased the ability of cMSCs to attract monocytes compared with untreated (Figure 3f). Our transcriptomics analysis revealed enrichment of the “response to IFN‐β” pathway (Figure S4a), and IFN‐β has been previously described as a cytokine involved in paracrine senescence (Yu et al., 2015). Thus, we compared the responses to IFN‐β versus IL‐1ß. While IFN‐β treatment up‐regulated expression of *Irf7*, a type I interferon‐response gene, it did not affect expression of the senescence‐associated genes (*Cdkn2a, Cdkn2b*) nor the SASP chemokines (*Ccl2, Ccl8*) (Figure S4c–e). These data revealed that cMSCs are prone to paracrine senescence in response to IL‐1ß but not IFN‐ß. Interestingly, treatment with IL‐1ß, but not IFN‐ß, decreased the expression of CD90, a classical marker of the mesenchymal lineage, at both the mRNA and protein levels (Figure 3g–i and Figure S4f).

To determine whether IL‐1ß could exert similar effects on human cells, cMSCs from cardiac biopsies of patients with advanced heart failure, characterized by expression of CD73, CD29 and CD140a (Figure S5a), were treated in vitro by IL‐1ß.

IL‐1ß also decreased proliferative activity (% Ki67+) (Figure 3j,k) of human cMSCs and increased expression of the main SASP components (*CCL2, IL6, IL8, VEGFA*) (Figure 3l). Down‐regulation of CD90 was also observed after IL‐1ß treatment for human cMSCs (Figure 3m–o). Hence, these results identified IL‐1ß as a potent mediator of paracrine senescence for both murine and human cMSCs, which also decreased the expression of CD90 in cMSC population.

### 2.4 Loss of CD90 expression is a hallmark of cMSC aging

**Figure 4:**
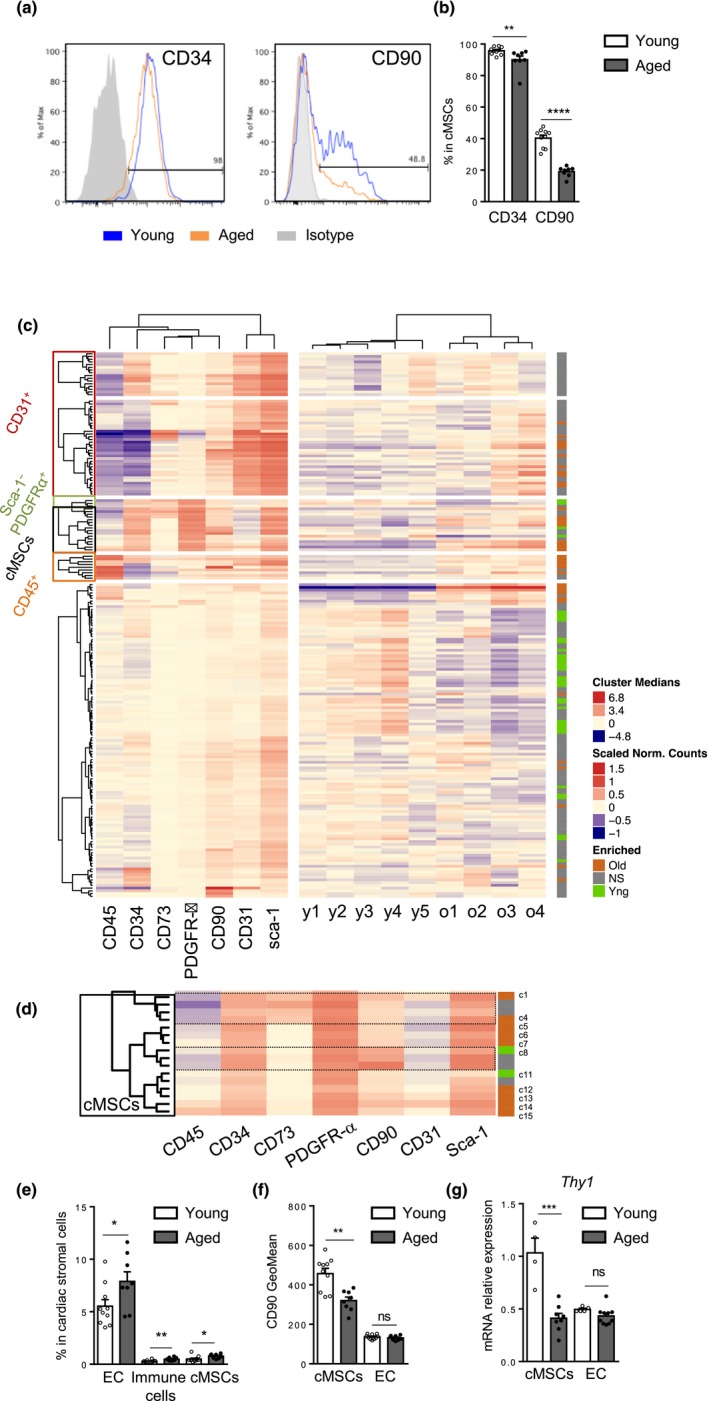
Loss of CD90 expression is a hallmark of cMSC aging. (a–b) Representative histograms (a) and percentages (b) of CD34 and CD90 in young (blue) and aged (orange) cMSCs, and isotype control (gray) (*n* = 8–10 mice per group). (c–d) Heatmap representation of DECyt method (c). (Left) Median fluorescence intensity of surface markers per cluster. (Middle) Relative count per cluster of young (y1‐y5, *n* = 5) or aged (o1‐o4, *n* = 4) cardiac stromal cells from individual mice. (Right) Cluster significantly enriched in aged cells (orange) or in young cells (green). Enlargement of cMSC clusters (d). (e) Percentage of endothelial cells (EC, CD31+), immune cells (CD45+) and cMSCs in cardiac stromal cells by flow cytometry (*n* = 10 young and *n* = 8 aged mice). (f–g) Expression of CD90 at (f) the protein (GeoMean) or (g) relative mRNA (*Thy1*) levels in young and aged cMSCs or EC. Data are expressed as means ± *SEM*. *p < 0.05, **p < 0.01, ***p < 0.001, **** *p* < 0.0001 versus young group

To examine the phenotypic changes associated with aging in cMSCs, expression of several classical mesenchymal markers (Jones & Schafer, 2015; Mendez‐Ferrer et al., 2010; Pinho et al., 2013) was evaluated separately by flow cytometry. While expression of integrin‐αV (CD51), integrin‐ß1 (CD29), mEFSK4 and CD73 was not significantly modified with aging (Figure S5c,d), CD90 and CD34 expressions were decreased (Figure 4a,b).

In order to characterize cMSC subset diversity within cardiac stroma, we generated a new method named DECyt (Figure 4c). Hierarchical clustering of all cardiac stromal cells from young and aged mice was performed using 7 surface markers. Cells are partitioned into subpopulations (or clusters) by cutting the resulting tree at a specific height. Within each partition, the number of cells observed per mice is taken as a count matrix for use with DESeq (Love, Huber, & Anders, 2014) in order to find significant differential subpopulations. Results are shown in Figure 4c as side by side heatmaps summarizing, for each cluster, the fluorescence intensity of the markers (left), the relative count of cells per mice (middle), and the statistical significance between young and aged mice (right). Analysis of all cardiac stroma revealed that expression of some classical mesenchymal markers (Sca‐1, CD73, CD34, CD90) was also shared by other cell types such as endothelial and immune cells. Thanks to the hierarchical clustering, we could observe, within cardiac stroma, age‐related changes in cMSC as well as in EC (CD31+) and immune cell (CD45+) clusters confirmed by classical flow cytometry analysis (Figure 4c–e).

All cMSC clusters expressed CD34, whereas expression of CD90 or CD73 defined distinct subpopulations. Interestingly, expression of CD90 defined cMSC subsets specifically enriched in young cells as identified in cluster 8 (c8). In contrast, cMSC clusters with low levels of CD90 (c1, c4‐7, c12‐15) were significantly enriched with aging (Figure 4c,d). Loss of CD90 with aging was specific to cMSCs when compared to EC, as confirmed by classical analysis of CD90 GeoMean and *Thy1* gene expression (Figure 4f,g).

In conclusion, aging induced major changes in cardiac stromal cell diversity with a specific decrease of the CD90 subset in cMSC population.

### 2.5 CD90 expression identified a cMSC subset more prone to differentiate toward the endothelial cell lineage

**Figure 5:**
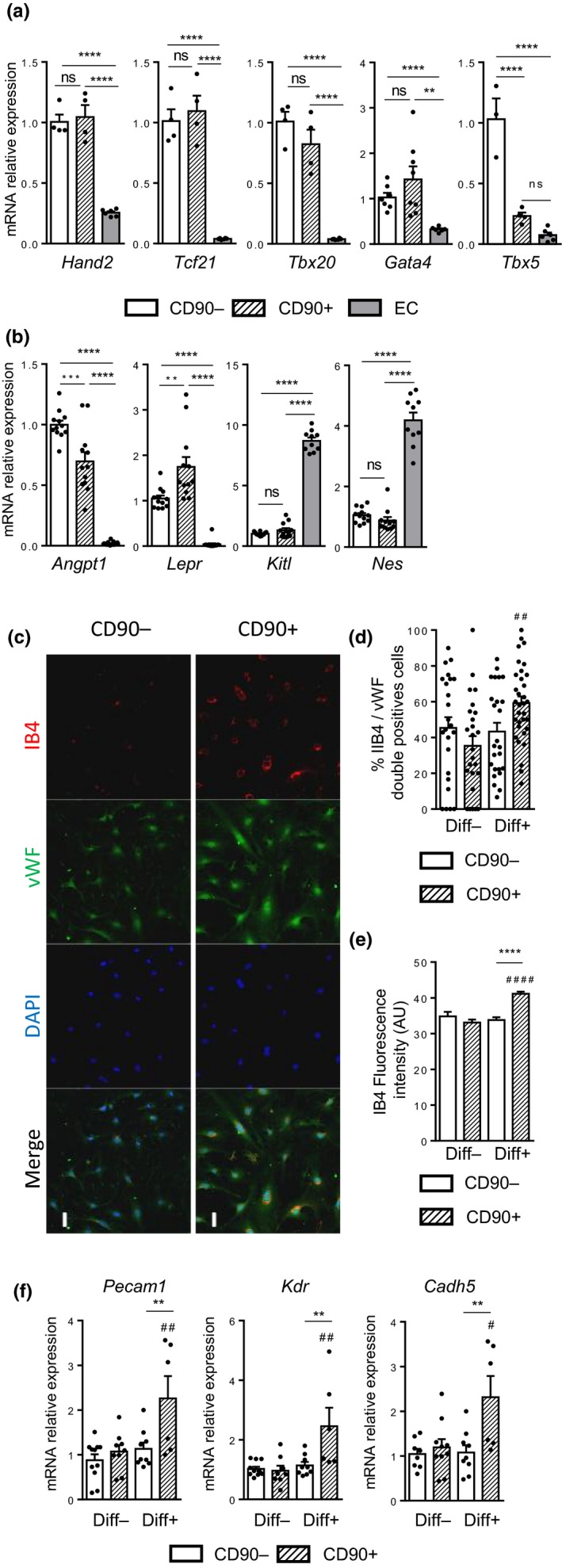
CD90+ expression defines a cMSC subset more prone to differentiate toward the endothelial cell lineage. (a‐b) Relative mRNA expression of pro‐epicardial (a) or stem cell niche (b) genes from young CD90‐ cMSCs (control group, CD90−, *n* = 4–13), CD90+ cMSCs (CD90+, *n* = 4–13), or endothelial cells (EC, *n* = 6–16). (c–f) Endothelial differentiation of young CD90+ or CD90‐ cMSCs. (c) Representative immunostaining with IB4 (red) and anti‐vWF (green) of CD90+ or CD90‐ cMSCs after culture with differentiation factors (nuclei: blue). Scale bar: 50 µm. (d) Percentage of differentiated cells (IB4 and vWF co‐expression) without (Diff‐) or with (Diff+) differentiation factors (*n* = 11 per group). (e) Fluorescence intensity of IB4 per cell (AU) per condition (*n* = 11 per group). (f) Relative mRNA expression of endothelial genes in CD90+ or CD90‐ cMSCs without (Diff‐) or with (Diff+) differentiation factors compared with Diff‐ CD90‐ (*n* = 6–11). Data are expressed as means ± *SEM*. (d‐f) §: Compared with CD90− Diff‐; #: Compared with CD90+ Diff ‐; * comparison between CD90‐ and CD90+ groups. *p < 0.05, **p < 0.01, ***p < 0.001, **** *p* < 0.0001

To verify that both the CD90‐ and CD90+ cMSCs belonged to the same cell population, we analyzed the expression of genes related to the cardiac mesenchymal lineage. Both CD90‐ and CD90+ cMSCs subsets from young mice expressed higher levels of *Tcf21*, *Tbx20*, *Tbx5*, *Hand2* and *Gata4* transcription factors compared with EC (Figure 5a), confirming the pro‐epicardial origin of both cMSC subsets (Chong et al., 2011; Noseda et al., 2015).

We then tested the expression of classical genes related to bone‐marrow (BM)‐MSC function and involved in the maintenance of the hematopoietic stem cell niche (Isern et al., 2014). *Kitl* and *Nes* had low expression in young CD90 + and CD90‐ cMSCs, whereas *Lepr* and *Angpt1* genes were significantly enriched in cMSCs compared with EC (Figure 5b). CD90+ cMSCs had higher *Lepr* expression than the CD90‐ subset. In contrast, expression of the *Angpt1* gene was higher in the CD90‐ cMSC subset (Figure 5b), suggesting a role of this subset in the regulation of angiogenesis through cross talk with ECs and stabilization of quiescent vessels (Carmeliet & Jain, 2011).

When the entire cMSC population from young and aged mice was stimulated with angiogenic factors for EC differentiation, *Thy1* mRNA (CD90) was up‐regulated (Figure S2i). However, these cells still expressed *Pdgfra* and *Ly6a* (Sca‐1) (Figure S2j) suggesting partial triggering of EC fate, resembling vasculogenic mimicry (Carmeliet & Jain, 2011).

We so compared young CD90+ and CD90‐ cMSC subsets for their vascular differentiation potentials. The CD90+ cMSC subset was characterized by an increased potency to differentiate toward the EC lineage in response to differentiation medium with higher fluorescence intensity of vWF and Isolectin B4 compared with the CD90‐ subset (2‐way ANOVA *p* = 0.01) (Figure 5c‐f). Moreover, in EC differentiation medium, only the CD90+ subset showed significant induction of *Cadh5* (2‐way ANOVA *p* = 0.027), *Pecam1* (2‐way ANOVA *p* = 0.04), and *Kdr* (2‐way ANOVA *p* = 0.0085) expression (Figure 5f). For SMC differentiation, we did not notice any significant difference between the two subsets except for the lower expression of αSMA protein in the CD90+ cMSCs (Figure S6a–d).

These data showed that CD90 expression helps to delineate a specific subset of cMSCs, with higher *Lepr* expression and more prone to acquire EC markers in response to VEGFA and FGF2.

### 2.6 Senescence of the cMSC CD90+ subset favored the acquisition of an endothelial‐like cell fate and correlated with the appearance of a new CD31+ cMSC subset in the aging heart

**Figure 6:**
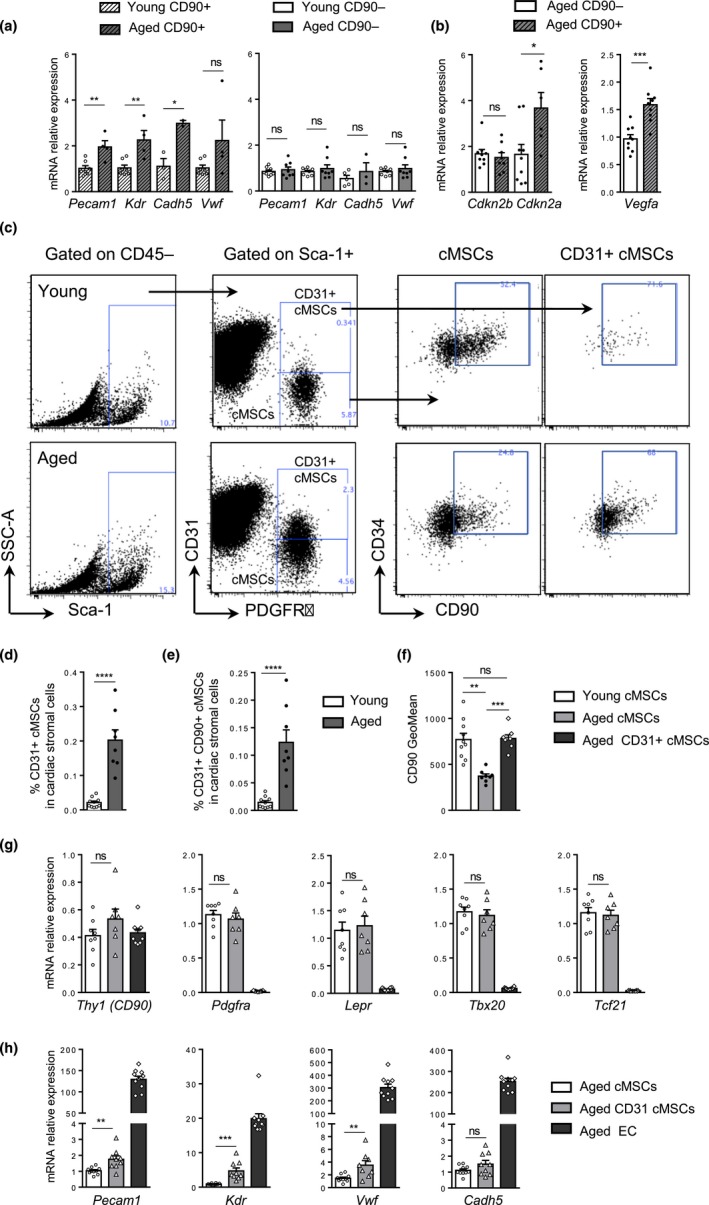
cMSC aging promoted acquisition of endothelial markers by the CD90+ subset and favored the emergence of a new CD31+ cMSC subset. (a) Relative mRNA expression of endothelial genes in young (*n* = 8) and aged (*n* = 8) CD90‐ (right) or CD90+ cMSC (left) subsets in vitro (control group: young CD90+). (b) Relative mRNA expression of CDKIs (left) or *Vegfa* (right) from ex vivo aged CD90+ (*n* = 9) or CD90‐ (*n* = 9) cMSCs (control group: young CD90‐, not shown). (c–f) Gating strategy (c) used to identify CD31‐ and CD31+ cMSCs by flow cytometry in cardiac stromal cells from young and aged mice (right) and dot plots of CD90 and CD34 in CD31‐ or CD31+ cMSCs (left). Histograms showed percentages of the CD31+ cMSCs (d) and of the CD90+ CD34+ CD31+ cMSC subset (e) in cardiac stromal cells from young (*n* = 10) and aged (*n* = 8) mice. (f) GeoMean of CD90 from young and aged CD31‐ cMSCs and from aged CD31+ cMSCs. (g‐h) Relative mRNA expression of mesenchymal (g) and endothelial (h) genes from ex vivo CD31‐ cMSCs (*n* = 8), CD31+ cMSCs (*n* = 8) and endothelial cells (*n* = 10) all from aged mice (control group: young CD31‐ cMSCs, not shown). Data are expressed as means ± *SEM*. *p < 0.05, **p < 0.01, ***p < 0.001, ****p < 0.0001

As we showed that aging decreased CD90+ cMSC frequencies, we asked whether aging could also impact the vascular differentiation potential of this subset. Young and aged CD90+ cMSC populations were cell‐sorted, cultured for 10 days without differentiation factors, and then tested for the acquisition of endothelial and smooth muscle cell markers. As shown in Figure 6a, aged CD90+ cMSCs expressed higher mRNA levels of *Pecam1, Kdr, Cadh5* and *Vwf* compared with young CD90+. No modulation of these genes could be detected for the CD90‐ subset (Figure 6a). On the contrary, aged CD90− cMSCs expressed higher mRNA levels of smooth muscle cell markers compared with young CD90−. No modulation or decreased expression of these markers was observed for the CD90+ subset (Figure S6e). Hence, these results showed that, with aging, the CD90+ and CD90‐ cMSC subsets spontaneously shifted in vitro toward two different cell fates, CD90+ cMSCs more prone to acquire EC markers and CD90‐ cMSCs, SMC ones.

Interestingly, ex vivo, aged CD90+ cMSCs had higher *Vegfa* expression compared with young and aged CD90‐ cMSCs (Figure 6b). Moreover, induction of *Cdkn2a* but not *Cdkn2b* was superior in aged CD90+ cMSCs than in aged CD90‐ cMSCs (Figure 6b). We previously observed that expression of *Cdkn2a* was up‐regulated during in vitro vascular differentiation of the entire cMSC pool and, notably, in aged cMSCs in response to endothelial differentiation medium (Figure S2j).

These results suggested that the preferential up‐regulation of *Cdkn2a* in the CD90+ cMSC subset with aging could favor, along with pro‐angiogenic factors such as *Vegfa*, the triggering of an endothelial differentiation program.

To test whether the acquisition of endothelial markers by aged CD90+ cMSCs could be observed in vivo, we performed flow cytometry analysis of cMSCs from young and aged mice without prior exclusion of CD31+ cells (Figure 6c‐e). Indeed, rare CD31+ cMSCs could be detected within the cardiac stroma of aged mice, which expressed CD34 and higher CD90 levels than aged conventional (CD31−) cMSCs (Figure 6f). This new CD31+ cMSC subset could be related to the c14 cluster identified in the DECyt heatmap, significantly up in aged mice and positive for CD31, CD34 and CD90 (Figure 4d). In order to better characterize this age‐related subset, the CD31+ cMSC, as well as the conventional (CD31‐) cMSC subset, and EC were cell‐sorted from aged mice and compared with young cMSCs (Figure 6g‐h). The CD31+ cMSC subset maintained signature gene expression of the mesenchymal lineage with similar levels of *Lepr*, *Pdgfra*, *Tbx20* and *Tcf21* as conventional (CD31−) cMSCs (Figure 6g). However, the CD31+ cMSC subset had higher gene expression of endothelial markers such as *Pecam1*, *Kdr* and *Vwf* (Figure 6h).

In conclusion, aging favors the acquisition of endothelial markers by the CD90+ cMSC subset and could contribute to the emergence in the cardiac microenvironment of a new cMSC subset expressing CD31 and other classical endothelial genes. This subset retained expression of mesenchymal‐related genes, suggesting partial commitment to an endothelial cell fate.

## 3 DISCUSSION

In diverse organs, a better understanding of stroma heterogeneity and locally produced microenvironmental factors has revealed key roles for non parenchymal cells in tissue homeostasis (Gude, Broughton, Firouzi, & Sussman, 2018). Of the structural modifications classically observed in the aging heart, such as perivascular fibrosis, cardiomyocyte hypertrophy, microvascular rarefaction, and increased tortuosity of coronary vessels (Han, 2012; Pries et al., 2015), many are caused by stroma cells or interactions between stroma and parenchymal cells. Biology of some tissue‐specific progenitor cells in the human heart has been shown to be affected by aging and, by undergoing cellular senescence and acquisition of SASP, could have deleterious impact on cardiac homeostasis and repair during aging (Lewis‐McDougall et al., 2019). The development of new strategies to combat cardiovascular diseases in the elderly will depend on a better understanding of how aging modulates the cardiac microenvironment.

We have demonstrated that aging strongly impacts cMSC biology through the modulation of specific functions, such as differentiation potential and paracrine activity. The increased expression of immune mediators, such as *Cxcl12*, *Cxcl13*, *Il33*, *Tnfsf13b,* and *Il7* with aging, endowed cMSCs with features of lymph node fibroblastic reticular cells (LN FRC) (Malhotra et al., 2012), revealed by a strong similarity with the stromal gp38+ CD31‐ CD140a+ LN FRC transcriptomic profile (Sample GSM777055 (GSE15907), Immgen.org *p*‐value = 8.465 e^−19^). Similar phenotypic changes have also been observed in a subset of colonic MSCs in the context of colitis (Kinchen et al., 2018). The acquisition of the FRC phenotype, during both aging and chronic inflammation, could confer an increased ability of MSCs to interact with immune cells.

Indeed, with aging, cMSCs, but not vascular ECs, up‐regulated *Ccl2* and *Ccl8* expression and increased the recruitment of monocytes by a CCR2‐dependent mechanism. This functional change correlated with an enrichment of monocyte‐derived CCR2+ macrophages in the cardiac MP pool. Previous reports have shown that the cardiac CCR2+ MP subset expresses IL‐1ß and is causally associated with pathological myocardial remodeling in both mice and humans (Bajpai et al., 2019, 2018), supporting that gradual changes in MP subsets with age could have a deleterious impact on the cardiac microenvironment. This hypothesis was strengthened by the increased production of pro‐inflammatory mediators (ROS, IL‐1ß) by the cardiac MP pool in the aging heart.

We hypothesized that MP‐derived IL‐1ß could in turn re‐enforces cMSC senescence. Microarray analysis supported this concept, as “response to cytokine” was one of the main biological process identified in aged cMSCs. We demonstrated that stimulation of cMSCs by IL‐1ß but not IFN‐ß mimicked several phenotypic and functional changes associated with aging in murine and human primary cell cultures. These results strongly support the concept that IL‐1ß production in the aging heart contributes to paracrine senescence of cMSCs and modulation of their subsets.

The DECyt method enabled us to visualize several age‐dependent modifications in the composition of the cardiac stroma, impacting diverse populations and, in particular, decreasing the frequencies of CD90+ cMSCs. CD90 expression allowed us to delineate a specific cMSC subset, expressing *Lepr* and *Vegfa,* which preferentially acquired EC markers in response to differentiation medium.

CD90 is a well‐known marker of MSCs from diverse murine and human tissues (Jones & Schafer, 2015; Michelis et al., 2018); however, the role of this glycophosphatidylinositol anchored protein in mesenchymal cell biology is not yet fully understood. Gago‐Lopez *et al.* have shown that human cardiosphere‐derived cells expressing CD90 after long‐term culture had higher self‐renewing capacity and gave rise to a higher percentage of vWF positive cells in response to endothelial differentiation medium compared with CD90−. On the other hand, both subsets had similar SMC differentiation potential (Gago‐Lopez et al., 2014). Furthermore, inactivation of CD90 expression in human BM‐MSCs by shRNA has been shown to increase adipogenesis and chondrogenesis, arguing for a regulatory role of CD90 in the differentiation potentials of MSCs (Moraes et al., 2016).

Since we observed that the aged CD90+ cMSC subset was more prone to acquire EC markers in vitro, we hypothesized that aging could modulate the acquisition of an EC fate. This hypothesis was strengthened by the identification of a new cMSC subset arising with aging in the cardiac microenvironment, expressing intermediate levels of CD31 and CD90. This subset also transcriptionally expresses other classical endothelial markers such as *Vwf* and *Kdr*, albeit to a lesser extent than ECs. These non conventional CD31+ cMSCs retained expression of transcription factors of pro‐epicardial origin (*Gata4*, *Tcf21*), along with PDGFR‐α, suggesting that they have partially converted to an endothelial cell fate, resembling vascular mimicry (Carmeliet & Jain, 2011).

At the molecular level, the CD31+ cMSC subset had strong similarities with endovascular progenitors (EVPs) involved in developing vasculature during wound healing (Patel et al., 2017) and tumor growth (Donovan et al., 2019). Similar to CD31+ cMSCs, EVPs express Sca‐1, PDGFRα, CD34 and CD90, as well as low to intermediate levels of endothelial markers. The generation of ECs from EVPs has been shown to go through a transitional stage, dependent on Notch (Heyl) signaling, but independent on VEGFA signaling (Donovan et al., 2019). Our microarray data showed that expression of *Heyl* and *Hey1* was reduced in aged cMSCs which could restrain their ability to fully differentiate toward the EC lineage and could explain the increased frequency of CD31+ cMSCs. Understanding how aging leads to modulation of the Notch pathway in CD31+ cMSCs, prohibiting their full conversion to the EC fate, requires further investigation. We currently hypothesize that the reduction of CD90+ cMSCs is due to both IL‐1ß‐dependent paracrine senescence and conversion to non conventional CD31+ cMSCs, blocked at a transitional stage with EC‐like features. The age‐related modifications that take place within the cardiac microenvironment include alterations in cMSC subset dynamics and senescence‐associated functional changes.

Modifications of the cardiac microenvironment are known to impact parenchymal cell function and contribute to the incidence of numerous cardiac diseases. We have shown here that aging leads to negative effects, exerted by the stroma, on cardiac homeostasis. Genetic and environmental factors likely exacerbate these natural tendencies leading to a higher frequency of cardiac disease among the elderly.

## 4 EXPERIMENTAL PROCEDURES

A more detailed account of experimental procedures can be found in the Supplementary Material [Link] accompanying this article**.**

### 4.1 Primary cardiac cell isolation

For murine cardiac cells, hearts were harvested from young mice (4 months ± 1.47) and aged mice (20 months ± 2.55) and enzymatically processed as indicated in Appendix S1. Cardiac stromal cells were stained with specific fluorochrome‐conjugated mAbs and cell‐sorted by high speed sorting with BD InfluxTM cell sorter (BD Biosciences). cMSCs were identified based on CD45− CD31− Sca‐1+ PDGFR‐α+ expression, macrophages on CD45+ CD64+ MHCII+, and vascular ECs on CD45− PDGFRα‐ CD31+ Sca‐1+ expression.

Human MSCs (hMSCs) were extracted from apex biopsies by enzymatic digestion and selected by overnight adhesion and cultures as indicated in Appendix S1.

### 4.2 Vascular differentiation of cMSCs

Cell‐sorted cMSCs were seeded in 24‐well plates coated with gelatin (0.75%) for RNA analysis and in 8‐well culture chamber slides (Lab‐Tek II, Nunc) for immunofluorescence assays. For EC differentiation, cMSCs were cultured for 10 days with 10 ng/ml VEGFA (Peprotech), 10 ng/ml FGF2 (Peprotech), and blocking anti‐human/mouse TGF‐β antibody (clone 19D8) at 5 μg/ml (Biolegend) in EGM2 media supplemented with 2% FCS and 1% Penicillin/Streptomycin (Promocell). For SMC differentiation, cMSCs were cultured for 10 days with 50 ng/ml TGF‐β1 (Peprotech) and 50 ng/ml PDGF‐BB (Peprotech) in DMEM (GIBCO) 10% HIFCS (Sigma) and 1% Penicillin/Streptomycin (Sigma).

Control medium consisted of EGM2 with 2% FCS but without growth factors, supplements, or anti‐TGFß. These assays were started directly after cell sorting, at P0.

### 4.3 Cytokine treatments

cMSCs were treated three times (every 2 days) with recombinant IL‐1β or IFN‐β at 10 ng/ml (Peprotech) in αMEM 10% HIFCS. Eight days after the first treatment, cells were either lysed for mRNA extraction or stained for flow cytometry. These assays were started at P0 or P1, and the flow cytometry analysis was performed after cell trypsinization.

### 4.4 Microarray

Gene expression microarrays were realized with Agilent SurePrint G3 Mouse GE v2 8x60K, design 074,809 (accession number GSE129656). RNA was extracted from cMSCs (pool of 3 mice), and quality and dosage were controlled with a Fragment analyzer (Advance Analytical). After Feature Extraction (Agilent), data were analyzed in R using the limma package (www.r-project.org, R v. 3.0.1, www.bioconductor.org v 2.12, limma v2.34.9). The data have been deposited in NCBI's Gene Expression Omnibus (Edgar *et al*., 2002) and are accessible through GEO Series accession number GSE129656 (https://www.ncbi.nlm.nih.gov/geo/query/acc.cgi?acc=GSE129656).

GO Biological Processes were identified using the ToppGene website with genes that limma found to be differentially expressed between young and aged samples with a *p* value < 0.01 and an absolute Log_2_ FC > 0.5 (https://toppgene.cchmc.org/prioritization.jsp).

### 4.5 Statistics

Results are expressed as means ± SEM. The statistical significance between two groups was estimated using either unpaired Student's two‐tailed *t* test or with the nonparametric Mann–Whitney U test, when indicated in the figure legend. Interactions between the effect of age and the subset of cMSCs used for vascular differentiation were evaluated with 2‐way ANOVA. Multi‐group comparisons were performed using either 1‐way ANOVA with Bonferroni's post hoc test or, when indicated, with the Kruskal–Wallis test with Dunn's post hoc test, for samples with a nongaussian distribution. Differences between groups were tested using GraphPad Prism software (version 7) and considered statistically significant for *p* < 0.05.

## AUTHORS’ CONTRIBUTION

M.H, L.L, I.JS, and D‐E.V designed, performed research, analyzed the data, and wrote the paper. I.JS and D‐E.V developed the DECyt method. M.DJ, M.D, I.R, and D.M performed experiments and analyzed the data. R.J and D.C collected the cardiac biopsies, designed the experiments, and reviewed the manuscript. P.N helped to design experiments. M‐P. J, C.D, and P.A helped to design the research and reviewed the manuscript.
