# Supplementary material for: Aging induces cardiac mesenchymal stromal cell senescence and promotes endothelial cell fate of the CD90 + subset
Source: Aging Cell. 2019 Jul 29;18(5):e13015. doi: 10.1111/acel.13015 (PMC6718537; doi:10.1111/acel.13015)
Supplement: Supplementary file 1 [file ACEL-18-e13015-s001.pdf]

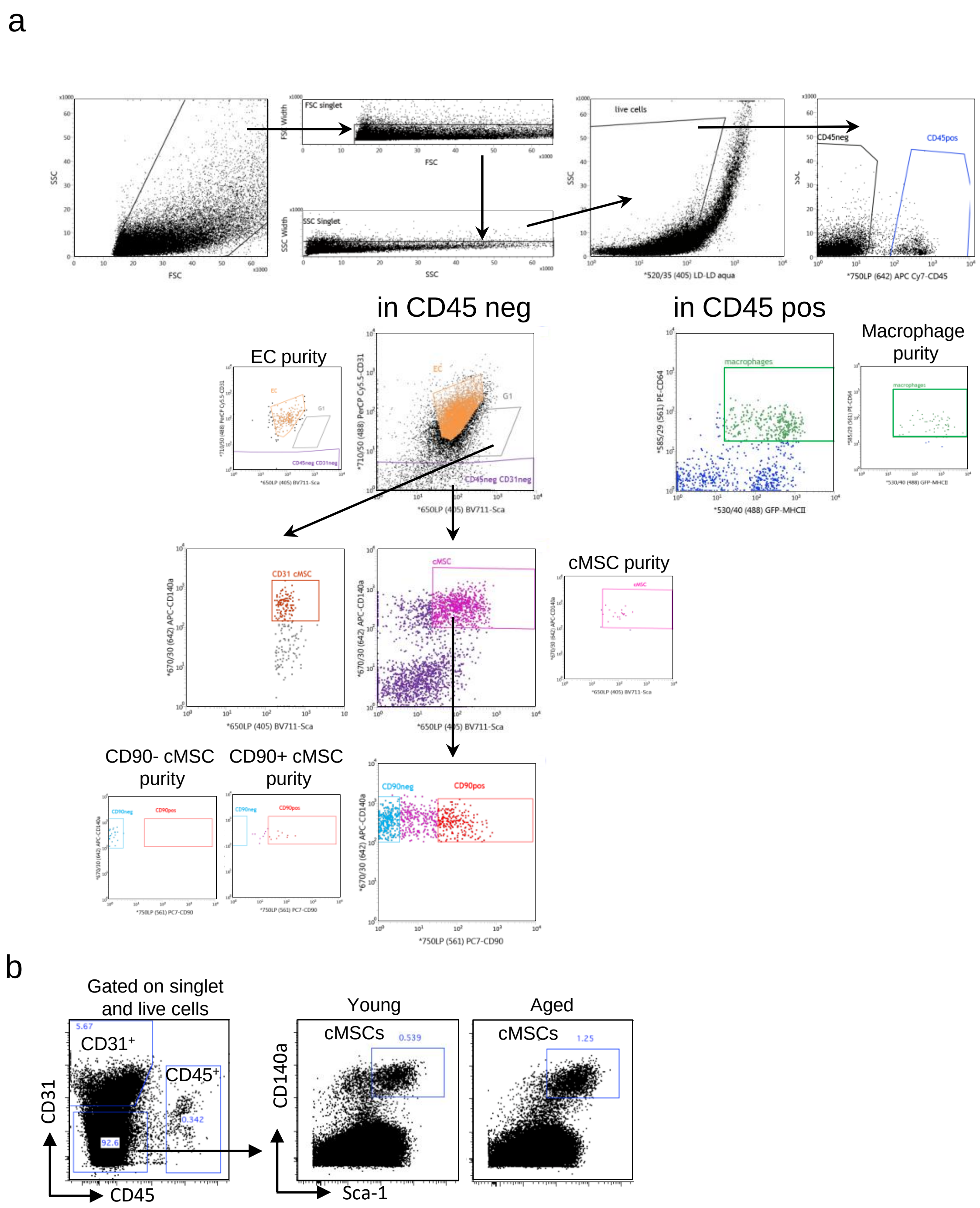

Supplemental Figure 1 : Cell sorting gating strategy

a: Representative gating strategy of cell sorting to isolate the populations of interest. Cardiac stromal cells were first selected on singlets (SSCA, SSCW) and live cells (aqua live dead negative, SSCA).

cMSCs were selected based on the negative expression of CD45 (SSCA, CD45), negative expression of CD31 (Sca-1, CD31), positive expression of CD140a and Sca-1 (CD140a, Sca-1). CD90+ cMSCs (red) and CD90- cMSCs (blue) were isolated based on CD90 levels and CD140a.

CD31+ cMSCs were selected as cells with intermediate levels of CD31 and high Sca-1 expression (Sca-1, CD31), and positive for CD140a and Sca-1 (CD140a, Sca-1).

Vascular endothelial cells (EC) were selected based on the negative expression of CD45 (SSCA, CD45) and positive co-expression of CD31 and Sca-1 (Sca-1, CD31).

Cardiac macrophages were positive for CD45 (SSCA CD45), positive for CD64 and expressed low to high levels of MHCII (CD64, MHCII).

Examples of purity rate are shown.

b: Representative dot-plots of CD140a and Sca-1 staining in CD45- CD31- cardiac stromal cells used to identify young and aged cMSCs by flow cytometry.

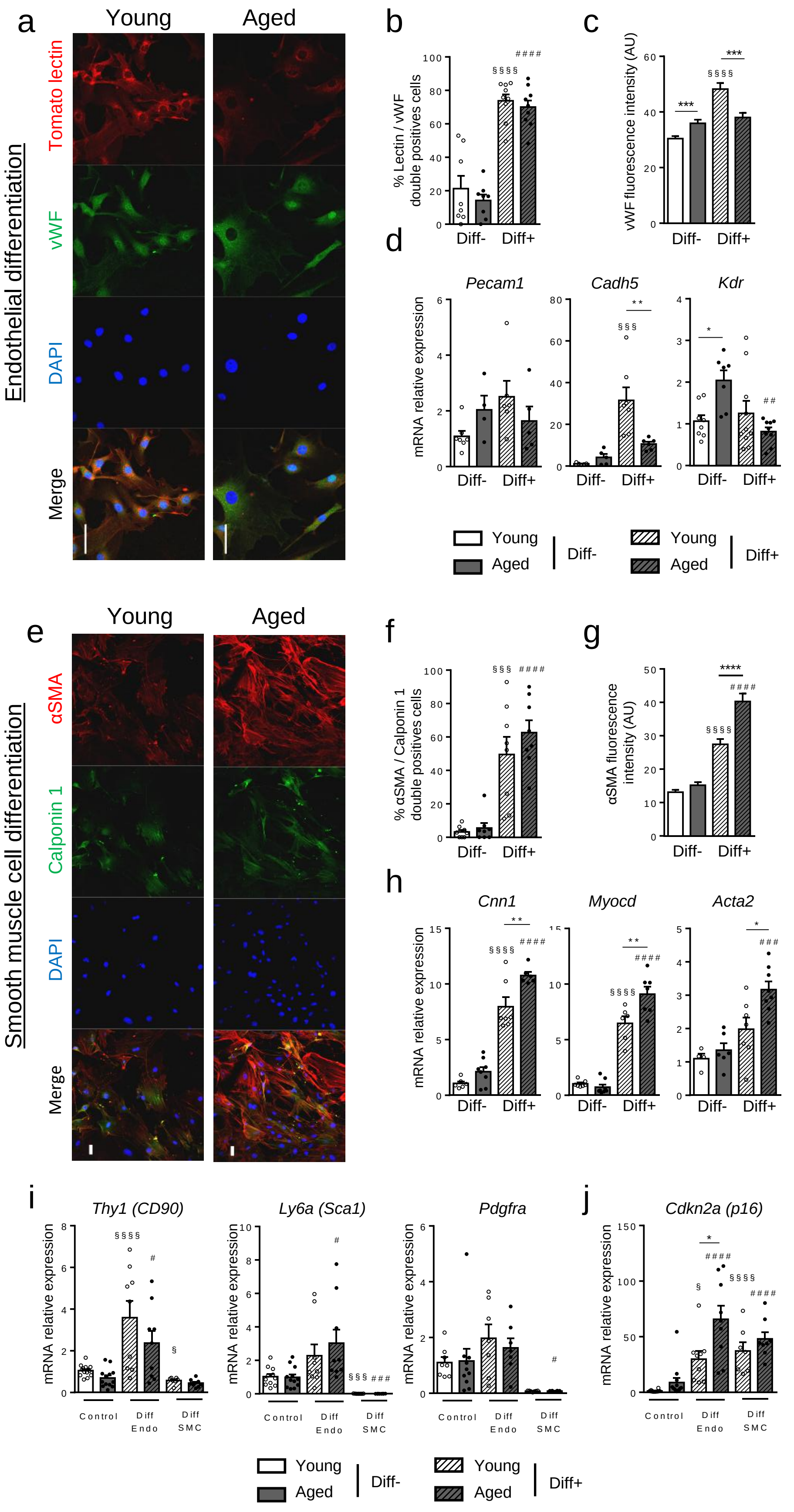

Supplemental Figure 2: Age impacted cMSCs vascular differentiation potential.

a-d: Endothelial differentiation of young and aged cMSCs. (**a**) Representative immunostaining with Tomato lectine (red), vWF (green), nuclei (DAPI, blue) of cMSCs with differentiation factors (Diff +). Scale bar: 50 µm (**b**) Percentage of differentiated cells (Tomato lectin and vWF co-expression) with (Diff+) or without (Diff-) differentiation factors (n=8 per group) (**c**) Fluorescence intensity of vWF per cell (UA) per condition (**d**) Relative mRNA expression of endothelial genes compared to young Diff- cMSCs.

e-h: Smooth muscle cell differentiation of young and aged cMSCs (**e**) Representative immunostaining with αSMA (red), Calponin 1 (green) of cMSCs with differentiation medium (Diff +). Nuclei, DAPI (blue). Scale bar: 50 µm. Percentage (**f**) of differentiated cells (αSMA and Calponin 1 co-expression) with (Diff+) or without (Diff-) differentiation medium and fluorescence intensity (**g**) of αSMA per cell (AU) per condition. Relative mRNA expression (**h**) of smooth muscle genes compared to young Diff- cMSCs (n=8 per group). **i-j**: Relative mRNA expression of mesenchymal genes (**i**) and *Cdkn2a* (**j**) compared to young Diff- cMSCs (n=8 per group).

Data are expressed as means ± SEM and were analyzed by ANOVA test.

§: compare to young Diff- ;

#: Compare to age Diff - ;

* comparison between young and aged groups *p < 0.05, **p < 0.01, ***p < 0.001, **** p < 0.0001.

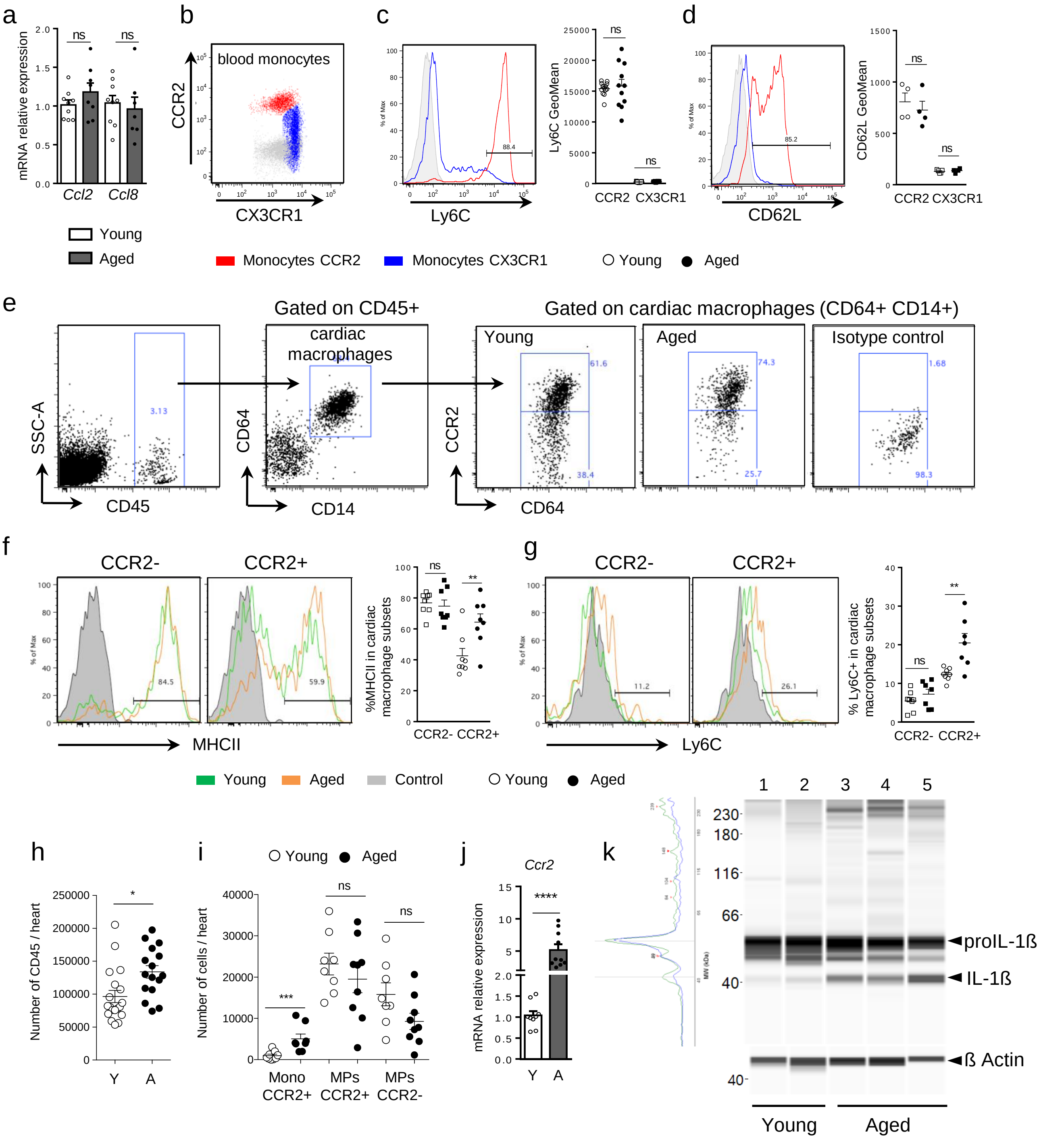

Supplemental Figure 3: Analysis of blood monocytes and cardiac macrophage phenotypes with aging.
a: Relative mRNA expression of *Ccl2* and *Ccl8* from aged endothelial cells compared to young (n= 9 young and n=8 aged mice) **b-d**: Blood monocyte subsets with aging. Dot plot representation (**b**) of CCR2 (red) and CX3CR1 (blue) expression by monocyte subsets using flow cytometry. Histogram (left) and geometric mean fluorescence intensity (right) of Ly6C (**c**) or CD62L (**d**) in CCR2 (red) or CX3CR1 (blue) blood monocyte subsets from young (n=13) or aged (n=11) mice. **e**: Representative gating strategy to analyze CCR2 expression by cardiac macrophages (cMPs) (CD64+ CD14+) in young and aged mice. Staining of CD14+ CD64+ cMPs with isotype control is shown (right). **f-g** Histogram (left) and percentages (right) of MHCII (**f**) and Ly6C (**g**) positive cells in CCR2+ or CCR2- cMP subsets from young (green) or aged (orange) mice (n=7-8 mice per group). **h** Number of CD45 cells per heart (n=15 young and n=12 aged mice). **i**: Number of CCR2+ monocytes, CCR2+ or CCR2- cMPs per heart (n=8 young and n=9 aged mice). **j**: Relative mRNA expression of *Ccr2* from aged cMPs compared to young (n=9 young and n=10 aged mice). **k**: Quantification of IL-1ß, pro-IL-1ß and ß-actin proteins in young or aged cMPs by western-blot using capillary electrophoresis. Proteins were extracted from young cMPs (pool of two mice per condition; lanes 1, 2) or aged cMPs (individual mice; lanes 3-5). Histograms (left) represented arbitrary units of chemiluminescence using anti-IL-1ß primary antibody.
Data are expressed as means ± SEM
*p < 0.05, **p < 0.01, ***p < 0.001, **** p < 0.0001 versus young group.

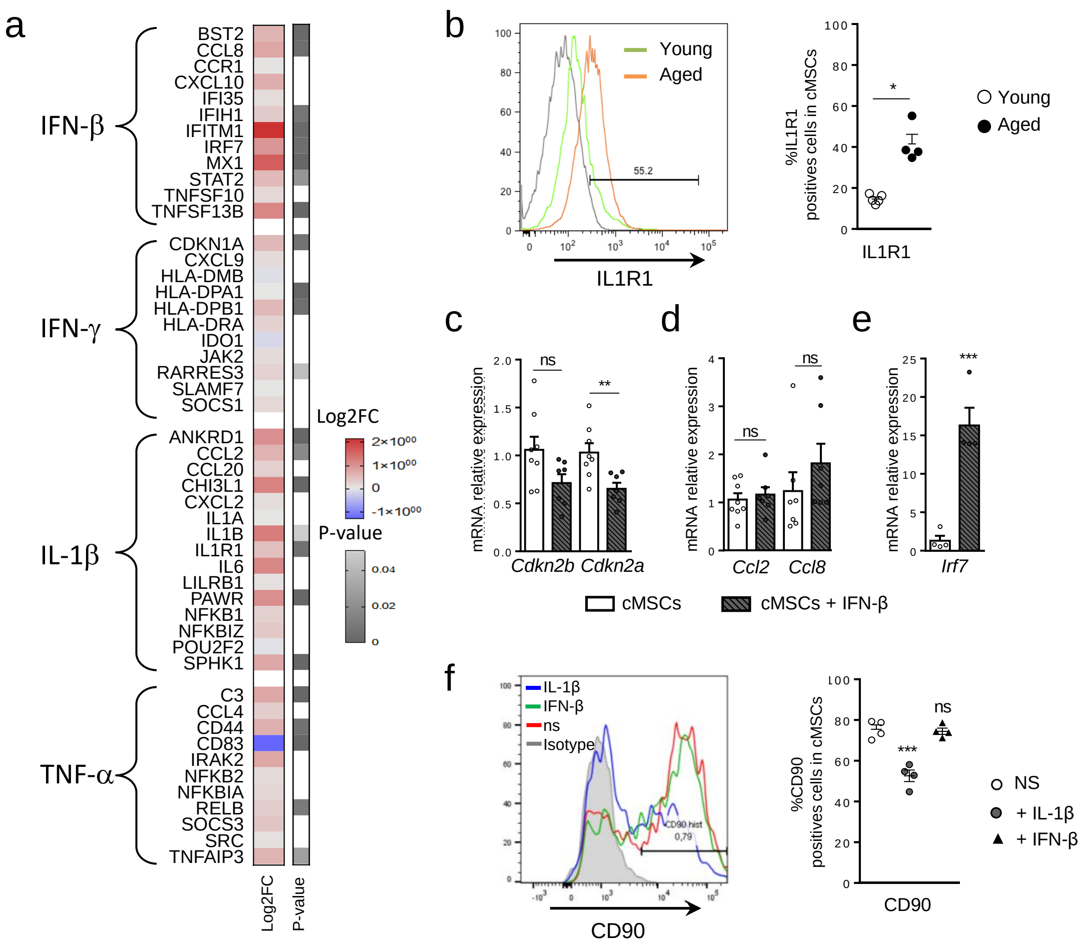

Supplemental Figure 4: Specific modulations of cMSC gene and CD90 expression in response to cytokine treatments

a: Log_2FC expression (left) of representative set of genes activated in response to cytokines (IFN-β, IFN-γ, IL-1β and TNF-α) from young and aged cMSCs. Data based on the microarray analysis. P-value (right) of the Log_2FC, in grey p-value < 0.05 **b**: Histogram (left) and percentage (right) of IL1R1 expression in young (n=4) or aged (n=4) cMSCs. **c-e**: Relative mRNA expression of CDKI (**c**), *Ccl2, Ccl8* (**d**) and *Irf7* (**e**) genes from IFN-β treated cMSCs compared to untreated cMSCs (n=4-8 per group). **f**: Histogram (left) and percentage (right) of CD90 expression in cMSCs untreated (ns, n=4), treated with IL-1ß (n=4) or with IFN-ß (n=4).

Data are expressed as means ± SEM. *p < 0.05, **p < 0.01, ***p < 0.001, **** p < 0.0001 versus untreated group.

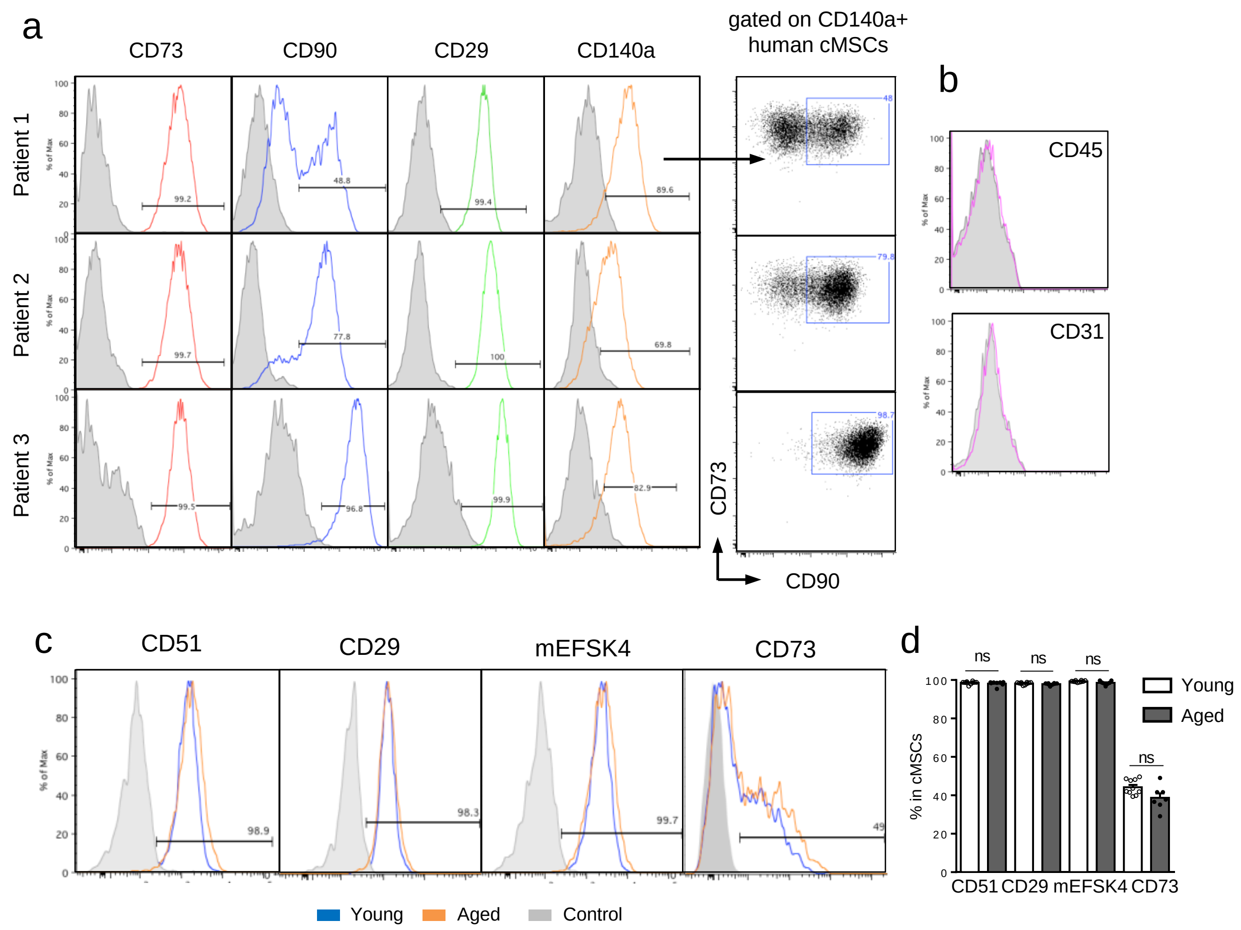

Supplemental Figure 5: Expression of mesenchymal markers by human and murine cMSCs
a: Examples of CD73, CD90, CD29 and CD140a expression compared to isotype controls (grey) by flow cytometry for human cardiac stromal cells isolated from cardiac biopsies (apex) of three patients. Dot-plot of CD90 and CD73 expression in CD140a+ human cMSCs for these patients. **b**: Histograms of human cMSCs showing negative expression for CD31 and CD45 by flow cytometry, overlay with isotype controls (grey).
c-d: Representative histograms (**c**) of CD51, CD29, mEFSK4 and CD73 expression by cMSCs from young (blue) and aged (orange) mice compared to isotype controls (grey) by flow cytometry. Percentages of positive cells (**d**) in young (n=10) or aged (n=7) cMSCs are indicated.
Data are expressed as means ± SEM. ns: not significant.

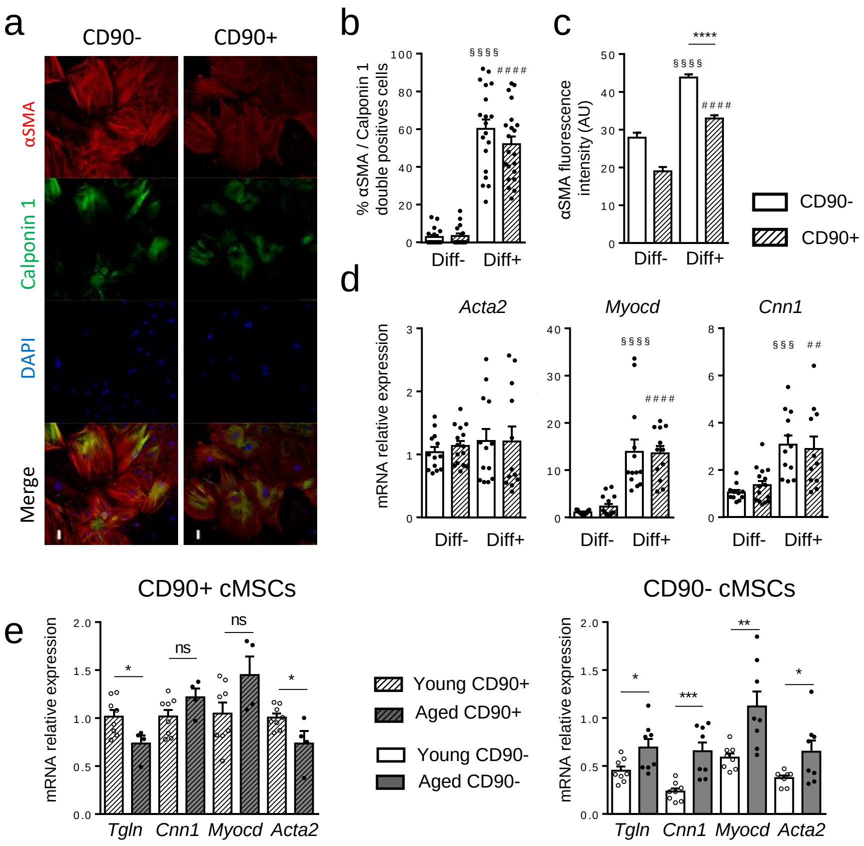

Supplemental Figure 6: Both CD90+ and CD90- cMSC subsets were prone to differentiate into smooth muscle cells.

a-d: Smooth muscle cell differentiation of young CD90+ or CD90- cMSCs cultured for 10 days in differentiation medium with or without differentiation factors (TGF-ß and PDGFBB). Representative immunostaining of cMSCs (**a**) with αSMA (red) and Calponin 1 (green), and nuclei (DAPI, blue) cultured with differentiation factors (Diff +) Scale bar: 50 µm. Percentage (**b**) of differentiated cells (αSMA and Calponin 1 co-expression) without (Diff-) or with (Diff+) differentiation factors and fluorescence intensity (**c**) of α-SMA per cell (AU) per condition (n=6-7 per group) (**d**) Relative mRNA expression of smooth muscle cell genes from young CD90+ or CD90- cMSCs *in vitro,* during the differentiation assay compared to control condition, CD90- without differentiation factors (Diff-) (n=11-15 per group). **e:** Relative mRNA expression of smooth muscle cells genes in young (n=8) and aged (n=8) CD90- (right) or CD90+ cMSC (left) subsets after culture for 10 days without TGF-ß and PDGFBB (control group: young CD90+).

Data are expressed as mean ± SEM and were analyzed by one way ANOVA.

§: Compare to CD90- Diff- ; #: Compare to CD90+ Diff - ; * comparison between CD90- and CD90+; *p < 0.05, **p < 0.01, ***p < 0.001, **** p < 0.0001.
