# Supplementary material for: Aging induces cardiac mesenchymal stromal cell senescence and promotes endothelial cell fate of the CD90 + subset
Source: Aging Cell. 2019 Jul 29;18(5):e13015. doi: 10.1111/acel.13015 (PMC6718537; doi:10.1111/acel.13015)
Supplement: Supplementary file 2 [file ACEL-18-e13015-s002.docx]

**Supplemental Materials and Methods**

**DECyt method**

DECyt was named as such because it permits one to apply a DESeq analysis on clustered cytometry data. The flowCore package (Hahne et al., 2009) was used to obtain R matrices from fcs files. The entire data set was clustered with Rclusterpp.hclust (Linderman, 2016) and cutree was applied to the resulting tree to partition cells into clusters. Each cluster was then summarized by both fluorescence parameter signature and number of cells per sample within the cluster. DESeq2 (Love, Huber, & Anders, 2014) was used with the cluster-count matrix in order to find clusters with significant differences between young and aged samples in terms of the counts of observations per sample per cluster.

Each cell, with its corresponding median fluorescence intensities, was extracted from FCS files and placed in rows of a matrix with functions from the flowCore package. Data was arc-sinh transformed using flowCore:arcsinhTransform( a = 0, b = 1/150 ). Each row was named with a sample specific label so that clusters could be converted to counts of cells per sample per cluster. The data from all samples were concatenated together and hierarchical clustering was applied to the complete data set with the hclust function from the Rclusterpp package. The resultant tree could then be partitioned into clusters by defining either the height (h) at which to cut or the desired number (k) of clusters. This step was seen to have a drastic influence on the results as a tree cut with insufficient resolution merged distinct subpopulations and a tree cut with too high of a resolution generated clusters with just a few cells per sample. In order to choose the best height for tree cutting, multiple heights were used to and the results were compared. The assignment vector from cutree was used to partition the data and each cluster was reduced to a median transformed fluorescence value as well as a count of the number of cells from each sample that were included in the cluster. The median signal of each parameter was used to assign each cluster a parameter signature. The count matrix was used with DESeq2 with a two group (young vs aged) design in order find clusters that were significantly different between the groups. Filtering clusters that had less than 100 cells per cluster in 60% of the samples for each group lead to the removal of less than 1% of the total cells per sample.

Heatmaps, from the ComplexHeatmap package, were generated from both the cluster parameter medians as well as the normalized, scaled count matrix. Regardless of whether or not the split parameter was used to break the heatmaps into blocks, the dendrogram on the left side shows the relationship between clusters in terms of their median signal signature. The top dendrogram above the central heatmap shows the relationship between the samples. Since we had employed a two group design in DESeq and therefore know both whether or not a cluster was significantly different and if it was more prevalent in aged or young samples, we added a third, single column heatmap to show this information by a three color code. In order to represent count data in a heatmap, we used the rlogtransformation function from DESeq to get a matrix of normalized counts and then scale each row to its mean.

**Animals**

The aging study was realized using male C57Bl/6JRj mice (Janvier Laboratories) and CX3CR1^gfp/+^ CD45.1 C57BL/6 heterozygote reporter mice. CX3CR1^gfp/+^ CD45.1 C57BL/6 mice, expressing eGFP under control of the endogenous Cx3cr1 promoter and harboring the CD45.1 allele, were bred in our animal facilities by backcrossing C57BL/6 CX3CR1^+/+^ CD45.1 males (Jackson laboratory) with C57BL/6 CD45.1 female mice (Charles River). Young mice (4 months +/- 1.47) and aged mice (20 months +/- 2.55) were used in the experiments.

All mice were maintained under specific and opportunistic pathogen-free conditions and handled according to procedures performed in accordance with the recommendations of the European Accreditation of Laboratory Animal Care (86/609/EEC) and guidelines established by the Ethics and Animal Safety Committee of INSERM Toulouse/ENVT (agreement number: C31 555 07).

**Preparation of cardiac stromal cell suspensions**

Hearts were harvested from mice after intraventricular perfusion of 10 ml PBS, minced with scalpels and digested with Liberase TM (Roche) diluted in RPMI1640 (GIBCO) as described previously (Laroumanie et al., 2014). Briefly, digestion of tissue fragments was performed by two successive incubations for 10 min with enzymatic solution at 37°C under shaking and stopped by the addition of heat-inactivated fetal calf serum (HIFCS) (SIGMA). Single cell suspensions were obtained by filtrations on 100 µm and 40 µm cell strainers (BD Falcon). Red blood cells were removed by hypotonic shock with ammonium chloride solution (0.83%) and cardiac stromal cells were washed and used for flow cytometry analysis or cell sorting.

**Human cardiac mesenchymal stromal cell cultures**

Apex biopsies were obtained from donor patients (men between the ages of 47 to 75 years, with a mean of 60.33) with advanced heart failure at the time of cardiac surgery for implantation of Left Ventricular Assist Device at the University hospital of Toulouse (France). Donor patients, with ischemic cardiomyopathy, signed informed written consent in accordance with the declaration of Helsinki and Local Ethics Committee approval (N° DC-2015-2456). Biopsies were minced and enzymatic digestion was performed using the same protocol as for murine hearts described above. Human cardiac ventricular MSCs (hMSCs) were selected by overnight adhesion and culture in αMEM (GIBCO) 10% HIFCS and 1% Penicillin/Streptomycin. Purity of hMSC cultures was assessed by flow cytometry (PDGFR-α+, CD29+, CD73+, CD31-, CD45-; Supplemental Figure 5a-b) and experiments were performed at passages P1 to P3.

**Cardiac stromal cell staining for flow cytometry and cell sorting**

Cells were incubated for 15 min at 4°C in blocking buffer as previously described (Laroumanie et al., 2014). Cells were then incubated on ice with conjugated monoclonal antibodies, diluted at their optimal concentrations (List 1), in FACS buffer (DPBS with 4% HIFCS, 2 mM EDTA). Necrotic cells were excluded by staining with Live/Dead Aqua or Yellow fluorescent reactive dyes (Life technologies).

Cardiac stromal cell subsets were isolated by high speed sorting with BD InfluxTM cell sorter (BD Biosciences) and cMSCs were cell sorted based on CD45- CD31- Sca-1+ PDGFR-α+ expression, macrophages on CD45+ CD64+ MHCII+ and vascular ECs were cell sorted on CD45- PDGFR-α- CD31+ Sca-1+ expression (Supplemental Figure 1).

**Clonogenic assays**

Cell sorted cMSCs from individual mice were seeded at 300 cells/well in 96 well-plates (12 wells per cMSCs) and cultured for 4 weeks in αMEM 10% FCS. Half the volume of medium was changed each week. Cells were then fixed with PFA 4% in DPBS, nuclei were stained with DAPI and 12 wells per condition were observed on an inverted fluorescent microscope (AXIOVision, Zeiss). CFU-fibroblasts (CFU-F) were count as positive if the number of cells per clone was equal or above 6. This assay was started directly after cell-sorting, at P0.

**Protein quantification by ELISA**

CCL2 concentration was quantified in 48h culture supernatants of cMSCs treated or not by IL-1β by ELISA (R&D System) following manufacturer’s instructions. The analyze was performed on a multimode spectra microplate reader (Infinite 200, Tecan) with the Magellan^TM^ software (Tecan).

**ROS quantification**

The oxygen-dependent respiratory burst of macrophages (ROS production) was measured after cell-sorting and adherence by chemiluminescence in the presence of 5-amino-2,3-dihydro-1,4-phthalazinedione (luminol, Sigma) using a thermostatically (37°C) controlled luminometer (Envision; Perkin Elmer). The generation of chemiluminescence was monitored continuously for 1 hour after incubation with luminol (66 mM) with or without 12-O-tetradecanoylphorbol-13-acetate (TPA, 100 µM). Statistical analyses were performed using the area under the curve expressed as counts per seconds.

**Immunofluorescence assays and confocal microscopy**

cMSCs were seeded on 8 well-culture chamber slides (Lab-Tek II, Nunc) at 10000 cells per well directly after cell-sorting and cultured for 3 days in αMEM 10% FCS. Adherent cMSCs were fixed with PFA 4% (Sigma-Aldrich) in DPBS. Cells were then incubated for 30 min in blocking buffer (DPBS with 5% FCS, 5% BSA and 0.2% Triton X100) and stained overnight at 4°C with primary antibodies (List 1) diluted in antibody buffer (DPBS with 2% FCS, 2% BSA and 0.2% Triton x100). After 3 washes in DPBS 0.02% Tween20 for 10 min, staining with secondary antibodies was performed for 1 hour in antibody buffer at RT. Nuclei were stained with DAPI (Invitrogen) for 10 min and slides were mounted with fluoromount G (Invitrogen). Images were acquired with an LSM 780 confocal microscope (Zeiss). Three to four images were acquired per wells and one image represented one field of acquisition (n=3-4 young and n=4 aged or IL-1β treated cMSCs per experiments). Images were analyzed with Fiji Software. For Ki67 and γH2AX stainings, results are expressed as percentage of positive cells per field of acquisition. For differentiation assays, results are expressed as percentage of double positive cells (Tomato lectin or Isolectine B4 and vWF, or αSMA and Calponin 1) per field of acquisition. Analyses of αSMA and vWF fluorescence intensities were performed on individual cells. The different immunofluorescence assays were performed at P0 after cell sorting and adherence.

**Chemotaxis assays**

Cell sorted cMSCs from individual mice were plated in 24 well-plates at 30000 cells per well and cultured in αMEM 10% FCS for 5 days. For chemotaxis assays with IL-1β, cells were treated as described before, and plated at 30000 cells per well in 24 well-plates after trypsinization. Treatment with IL-1β was maintained during the assay. Monocytes were isolated from murine bone marrow cells, after flushing of the tibia and femurs, using the EasySep™ Mouse Monocyte Isolation Kit according to manufacturer's protocol (Stem Cell #19861). Monocytes were labelled with CFSE (10 ng/ml) for 15 min and pre-incubated, or not, with CCR2 antagonist (10 µM, RS504393 Tocris) for 20 min. Monocytes (1.5 x 10^5^) were added to 3 µm pore size FluoroblokTM inserts (Corning) on top of either cMSCs or control medium in 24 well-plates. After 3 hours in the incubator at 37°C 5% CO_2_, Fluorobloks were fixed with PFA 4%, and monocyte nuclei were stained with DAPI. Only monocytes which have passed through the filter were quantified after imaging with LSM 780 confocal microscope (Zeiss). Four images were acquired and quantified per wells.

For the chemotaxis assays, young and aged cMSCs were tested at passage 0, after cell sorting and adherence. To test the effect of IL-1β treatment on cMSC chemotaxis activity, cMSCs, after cell sorting (P0), were treated by IL-1ß for 7 days and tested after their first passage.

**Protein quantification by capillary-based western blot**

A capillary-based "Simple Western System" (WES, ProteinSimple) was used to quantify IL1-β protein, according to the manufacturer’s protocol using goat anti mouse IL-1b/ IL-1F2 (AF-401-NA, R&D systems, 1/250) and normalized by ß-actin quantification using rabbit anti mouse ß-actin (4970, CTS, 1/250). Proteins were extracted from 50000 sorted cMPs using lysis buffer (Tris HCL 50 mM, NaCl 150 mM, NP40 1%, Deoxycholic Acid 1%, SDS 0.10%, EDTA 5 mM, NaF 20 mM, orthovanadate 2 mM and PMSF 2 mM) supplemented with protease and phosphatase inhibitor cocktails (Sigma). Protein concentrations were determined using a BCA Protein Assay Kit (Thermo Fisher Scientific) and adjusted to 0.15 mg/ml. Separation and immunoprobing were performed automatically and the chemiluminescent signals were detected and analyzed by Compass software (ProteinSimple).

**RT-PCR**

Total RNA was extracted using ReliaPrep™ RNA Miniprep Systems (Promega) and quantified by spectrophotometry (ND-100 NanoDrop, Thermofisher). cDNA was synthesized with either the MultiScribe™ reverse transcriptase (High-Capacity cDNA Reverse Transcription Kit, Applied Biosystem), or, for experiments with low amounts of RNA, with SuperScript™ VILO™ cDNA Synthesis Kit (Invitrogen). PCR was performed for 40 cycles with SYBR green (Takara) on a Viia7 (Thermo). Primer sequences were designed or checked using Primer-BLAST (Ye et al., 2012) and are listed in List 2. Rplp0 was used as a reference gene for normalization and relative gene expression, compared to the control group, was calculated using the comparative cycle threshold (CT) method (2^-∆∆CT^).

**References**

Hahne, F., LeMeur, N., Brinkman, R. R., Ellis, B., Haaland, P., Sarkar, D., . . . Gentleman, R. (2009). flowCore: a Bioconductor package for high throughput flow cytometry. *BMC Bioinformatics, 10*, 106. doi:1471-2105-10-106 [pii]

10.1186/1471-2105-10-106

Laroumanie, F., Douin-Echinard, V., Pozzo, J., Lairez, O., Tortosa, F., Vinel, C., . . . Pizzinat, N. (2014). CD4+ T cells promote the transition from hypertrophy to heart failure during chronic pressure overload. *Circulation, 129*(21), 2111-2124. doi:CIRCULATIONAHA.113.007101 [pii]

10.1161/CIRCULATIONAHA.113.007101

Linderman, M. (2016). Rclusterpp: Linkable C++ Clustering.

Love, M. I., Huber, W., & Anders, S. (2014). Moderated estimation of fold change and dispersion for RNA-seq data with DESeq2. *Genome Biol, 15*(12), 550. doi:s13059-014-0550-8 [pii]

10.1186/s13059-014-0550-8

Ye, J., Coulouris, G., Zaretskaya, I., Cutcutache, I., Rozen, S., & Madden, T. L. (2012). Primer-BLAST: a tool to design target-specific primers for polymerase chain reaction. *BMC Bioinformatics, 13*, 134. doi:10.1186/1471-2105-13-134

**List 1:** Antibodies

| Primary antibodies (Immunofluorescence) | | | |
| --- | --- | --- | --- |
| Target | Supplier | ref | |
| Senescence | | | |
| Ki67 | Cell Marque | 275R-14 | |
| γH2AX | Millipore | 05-636 | |
|  |  |  |  |
| Differentiation | | | |
| Tomato lectine | Vector | TL 1176 | |
| Isolectine B4 | Invitrogen | 121413 | |
| vWF | Dako | A0082 | |
|  |  |  | |
| αSMA | Sigma | A2547 | |
| Calponine (Cnn1) | Abcam | ab46794 | |
|  |  |  |  |
| Other | | | |
| DAPI | Sigma | D9542 | |

| Flow cytometry antibodies | | | | | | | | | |
| --- | --- | --- | --- | --- | --- | --- | --- | --- | --- |
| Cell sorting Panel | | | | |  | Panel cardiac macrophages | | | |
| Target | Clone | | Supplier | Ref |  | MHCII APC-Cy7 | M5/114.15.2 | Biolegend | 107627 |
| CD45 APC-Cy7 | 30-F11 | | Biolegend | 103116 |  | CCR2 PE | 475301 | R&D systems | FAB5538P |
| CD31 PerCP-Cy5.5 | 390 | | Biolegend | 102420 |  | CD64 APC | X54-5/7.1 | Biolegend | 139306 |
| Sca1 BV711 | D7 | | Biolegend | 108131 |  | Ly6C eFLuor450 | HK1.4 | eBioscience | 48-5932 |
| CD140a APC | APA5 | | Biolegend | 135908 |  | CD45 PE-Cy7 | 30-F11 | Biolegend | 103113 |
| CD90 PE-Cy7 | 5321 | | Biolegend | 140310 |  | Cx3cr1 gfp (mice) |  |  |  |
| CD64 PE | X54-5/7.1 | | Biolegend | 139303 |  | CD14 PerCP-Cy5.5 | Sa2-8 | eBioscience | 46-0141 |
| MHCII FITC | M5/114.15.2 | | Biolegend | 107605 |  |  |  |  |  |
|  |  | |  |  |  | Panel DECyt | | | |
| CD140a PE | APA5 | | Biolegend | 135905 |  | CD45 APC-Cy7 | 30-F11 | Biolegend | 103116 |
| Sca1 PB | D7 | | Biolegend | 108120 |  | CD140a PE | APA5 | Biolegend | 135905 |
|  |  | |  |  |  | CD34 APC | RAM34 | BD Pharmigen | 560233 |
| Yellow Dye |  | | Life technologie | L34959 |  | Sca1 FITC | E13-161.7 | Biolegend | 122505 |
| Aqua Dye |  | | Life technologie | L34966 |  | CD73 PE-Cy7 | TY/11.8 | Biolegend | 127223 |
|  |  | |  |  |  | CD90 PB | 53-2.1 | eBioscience | 48-0902-80 |
| Panel blood monocytes | | | | |  | CD31 PerCP-Cy5.5 | 390 | Biolegend | 102420 |
| Target | Clone | Supplier | | Ref |  |  |  |  |  |
| CD45 APC-Cy7 | 30-F11 | Biolegend | | 103116 |  | Others | | | |
| CCR2 PE | 475301 | R&D systems | | FAB5538P |  | mEFSK4 PE | mEFSK4 | Milteyi | 130-102-901 |
| CD43 APC | 1B11 | Biolegend | | 121213 |  | CD29 FITC | HMβ1-1 | Biolegend | 102205 |
| Ly6C eFluor450 | HK1.4 | eBioscience | | 48-5932 |  | IL1R1 PE | JAMA-147 | Biolegend | 113505 |
| CD62L PE-Cy7 | MEL-14 | eBioscience | | 104417 |  | CD51 PE | RMV-7 | Biolegend | 551187 |
| Cx3cr1 gfp (mice) |  |  | |  |  | CD51 PE | RMV-7 | Biolegend | 551187 |
| CD14 PerCP-Cy5.5 | Sa2-8 | eBioscience | | 46-0141 |  |  |  |  |  |
|  |  |  | |  |  |  |  |  |  |
|  |  |  | |  |  |  |  |  |  |
| Human | | | | |  |  |  |  |  |
| CD31 PerCP | WM59 | Biolegend | | 303131 |  |  |  |  |  |
| CD45 APC-Cy7 | HI30 | Biolegend | | 304014 |  |  |  |  |  |
| CD140a PE | 16A1 | Biolegend | | 323505 |  |  |  |  |  |
| CD90 BV421 | 5E10 | Biolegend | | 328121 |  |  |  |  |  |
| CD73 PE-Cy7 | AD2 | Biolegend | | 344009 |  |  |  |  |  |
| CD29 AF488 | TS2/16 | Biolegend | | 303015 |  |  |  |  |  |

**List 2**: Primers

| **Primers (Mouse)** | | | | | | |
| --- | --- | --- | --- | --- | --- | --- |
| *Acta2* | S | TCCCTGGAGAAGAGCTACGAA |  | *Il1rn* | S | ACATGGCAAACAACACAGGA |
|  | AS | TATAGGTGGTTTCGTGGATGCC |  |  | AS | TAGCAAATGAGCCACAGACG |
| *Angpt1* | S | CAGCATCCTGCAGAAGCAAC |  | *Il6* | S | GAGGATACCACTCCCAACAGACC |
|  | AS | TCCTCCCTTTAGCAAAACACC |  |  | AS | AAGTGCATCATCGTTGTTCATACA |
| *Arg1* | S | AGAGCTGACAGCAACCCTGT |  | *Irf7* | S | CCAGCTCTCACCGAGCG |
|  | AS | GGATCCAGAAGGTGATGGAA |  |  | AS | TACAGGAACACGCATCTGGG |
| *Cadh5* | S | CCCCAAACGTAACGAGGGTAT |  | *Itgam* | S | CTCCCAAAGTGCTGGGATTA |
|  | AS | TCGTATCGGATAGTGGGGTCT |  |  | AS | TTGCATCCATCTCAAATCCA |
| *Ccl2* | S | AAACCTGGATCGGAACCAAAT |  | *Kitl* | S | CCCTGAAGACTCGGGCCTA |
|  | AS | TACGGGTCAACTTCACATTCAAA |  |  | AS | CAATTACAAGCGAAATGAGAGCC |
| *Ccl8* | S | CACCTGAGTTAAGAGACAGCCAAAG |  | *Kdr* | S | GAACAGTAAGCGAAAGAGCCG |
|  | AS | TGAGAAAACACGCAGCCCA |  |  | AS | TGTCTGTCTGGCTGTCATCTG |
| *Ccr2* | S | CCTTGGGAATGAGTAACTGTGTGA |  | *LepR* | S | TGATGTGTCAGAAATTCTATGTGG |
|  | AS | AATGACAGGATTAATGCAGCAGTGT |  |  | AS | TGCCAGGTTAAGTGCAGCTAT |
| *Cd163* | S | CATCATGGCACAGGTCACCC |  | *Mmp3* | S | TTGATGGGCCTGGAACAGTC |
|  | AS | CGCTGAATCTGTCGTCGCTT |  |  | AS | GGTTGGTACCAGTGACATCCT |
| *Cd68* | S | GCCCAAGGAACAGAGGAAGACT |  | *Myocd* | S | GTTCAGCTACCCTGGGATGCACCA |
|  | AS | GTAGGGCTGGCTGTGCTTTCT |  |  | AS | GGCCTGGTTTGAGAGAAGAAACAC |
| *Cdkn1a* | S | GCAGAATAAAAGGTGCCACAGGC |  | *Nestin* | S | AGCAGGGTCTACAGAGTCAGA |
|  | AS | CCGAAGAGACAACGGCACACT |  |  | AS | CCTCCAGCAGAGTCCTGTATG |
| *Cdkn2a* | S | CCGAACTCTTTCGGTCGTACCC |  | *Nos2* | S | ACCACTCGTACTTGGGATGT |
|  | AS | CTGCTACGTGAACGTTGCCCA |  |  | AS | CACCTTGGACTTCACCCAGT |
| *Cdkn2b* | S | AGATCCCAACGCCCTGAAC |  | *Pdgfra* | S | ACTTTTCACTCCGGGTATCGG |
|  | AS | CAGTTGGGTTCTGCTCCGT |  |  | AS | CTGAGGACCAGAAAGACCTGG |
| *Cdkn2c* | S | GGGGGACCTAGAGCAACTTAC |  | *Pecam1* | S | AGCCAACAGCCATTACGGTTA |
|  | AS | CTCCGGATTTCCAAGTTTCA |  |  | AS | CTCAAGGGAGGACACTTCCAC |
| *Chi3l3* | S | AATGATTCCTGCTCCTGTGG |  | *Postn* | S | GGACACCTCGTGGCAGTTTC |
|  | AS | ACTTTGATGGCCTCAACCTG |  |  | AS | CCGCCATCACCTCTGACCT |
| *Cnn1* | S | ACGGCTTGTCTGCTGAAGTAA |  | *Sca1* | S | GTTTGCTGATTCTTCTTGTGGCCC |
|  | AS | TGAGGCCATCCATGAAGTTGT |  |  | AS | ACTGCTGCCTCCTGAGTAACAC |
| *Cx3Cl1* | S | TGGCTTTGCTCATCCGCTATCAG |  | *Sod1* | S | TGTGCGTGCTGAAGGG |
|  | AS | CGTCTGTGCTGTGTCGTCTCC |  |  | AS | CATACTGATGGACGTGGAAC |
| *Fli1* | S | AATGTGTGGAATATTGGGGG |  | *Tagln* | S | ACCAAAAACGATGGAAACTACCG |
|  | AS | GGACTGATCGTCACTCACCA |  |  | AS | CATTTGAAGGCCAATGACGTG |
| *Gata4* | S | CTGGAAGACACCCCAATCTC |  | *Tbx20* | S | CAGCCTACCAGAACCAACTGA |
|  | AS | CCATCTCGCCTCCAGAGT |  |  | AS | TCTCCACACTCTCCCTCTCAA |
| *Gdf6* | S | GGGCATCAATGCCAGCTTTT |  | *Tbx5* | S | CCCCTGTACAGAGCGAGAATA |
|  | AS | GTGCGAGAGATCGTCCAGTC |  |  | AS | GGCCAAAGCCCTCATCTGTAT |
| *Hand2* | S | CCTTCAAGGCGGAGATCAAGA |  | *Tcf21* | S | GGCCAACGACAAGTACGAGA |
|  | AS | CCTGTCCGGCCTTTGGTTTT |  |  | AS | GCTGTAGTTCCACACAAGCG |
| *Hgf* | S | TGCCCTATTTCCCGTTGTGAA |  | *Tgfb1* | S | ACTATTGCTTCAGCTCCACAGAGAA |
|  | AS | ACAACCCGCAGTTGTTTTGTT |  |  | AS | TGTACAGCTGCCGCACACA |
| *Il10* | S | TTCAGCCAGGTGAAGACTTTCT |  | *Thy1* | S | AACTCTTGGCACCATGAACC |
|  | AS | GCTTGGCAACCCAAGTAACC |  |  | AS | AGTCCAGGCGAAGGTTTTG |
| *Il12p40* | S | CGGACGGTTCACGTGCTC |  | *Tnfa* | S | CCACCACGCTCTTCTGTCTAC |
|  | AS | CACATGTCACTGCCCGAGAGT |  |  | AS | AGGGTCTGGGCCATAGAACT |
| *Il1a* | S | TTGGTTAAATGACCTGCAACA |  | *Vegfa* | S | CAGCAGATGTGAATGCAGACCAA |
|  | AS | GAGCGCTCACGAACAGTTG |  |  | AS | CTTTCTCCGCTCTGAACAAGGC |
| *Il1b* | S | GATCCACACTCTCCAGCTGCA |  | *Vwf* | S | TACAGCCCCTGTTTGTATGGG |
|  | AS | CAACCAACAAGTGATATTCTCCATG |  |  | AS | CTGTCACGGGGCTTTTCTGT |
|  |  |  |  |  |  |  |
| **Primers (Human)** | | | | | | |
| *CCL2* | S | TCCCAAAGAAGCTGTGATCTTCAA |  | *IL8* | S | GTGTGAAGGTGCAGTTTTGCCA |
|  | AS | TTTGCTTGTCCAGGTGGTCC |  |  | AS | TTGGGGTGGAAAGGTTTGGAG |
| *IL6* | S | TCAATGAGGAGACTTGCCTGGT |  | *VEGFA* | S | AACCATGAACTTTCTGCTGTCTT |
|  | AS | CACAGCTCTGGCTTGTTCCT |  |  | AS | ACTTCACCACTTCGTGATGATTCT |

**Supplemental Table**

**Supplemental Table 1: List of cardiac MSC genes significantly modified with aging from the microarray analysis.**

Differentially expressed genes from aged cMSCs compared to young, based on microarray analysis.

| Gene Symbol | Log_2_FC | p-value | Accession number |
| --- | --- | --- | --- |
| Hp | 4.177 | 1.62E-06 | NM_017370 |
| Serpina3g | 3.833 | 1.29E-05 | NM_009251 |
| S100a9 | 3.447 | 4.75E-04 | NM_001281852 |
| Lcn2 | 3.256 | 2.71E-04 | NM_008491 |
| Cfb | 3.054 | 1.39E-07 | NM_008198 |
| C4b | 2.647 | 7.85E-08 | NM_009780 |
| Nrk | -1.592 | 4.95E-08 | NM_013724 |
| Evl | -1.877 | 2.56E-09 | NM_001163394 |
| Rgs6 | -1.458 | 3.40E-08 | NM_015812 |
| Slc38a4 | -2.048 | 1.82E-07 | NM_027052 |
| Slc38a5 | -1.658 | 4.76E-03 | NM_172479 |
|  |  |  |  |
| Gm15433 [Csprs] | 2.393 | 1.76E-09 | XM_003084455 |
| Csprs | 2.393 | 1.81E-09 | NM_033616 |
| Dmkn | 3.066 | 3.75E-09 | NM_001166173 |
| Lgi2 | 2.108 | 4.18E-09 | ENSMUST00000039750 |
| ENSMUST00000160058 | 2.218 | 4.37E-09 | ENSMUST00000160058 |
| Lppr3 | 2.244 | 5.40E-09 | NM_181681 |
| C4b | 2.647 | 7.85E-08 | NM_009780 |
| Gm7609 [Csprs] | 1.555 | 8.13E-08 | NM_001081746 |
| Gm16026 | 1.555 | 9.85E-08 | ENSMUST00000111423 |
|  |  |  |  |
| Cdkn2a | 0.738 | 3.27E-05 | NM_009877 |
| Cdkn2b | 0.679 | 1.23E-03 | NM_007670 |
| Cdkn2c | 0.371 | 0.016973 | U19596 |
|  |  |  |  |
| Ccl2 | 0.604 | 0.012142 | NM_011333 |
| Ccl8 | 0.705 | 0.001898 | NM_021443 |
| Cx3cl1 | 1.6993 | 1.63E-07 | NM_009142 |
| Mmp3 | 1.297 | 2.70E-06 | NM_010809 |
| Postn | 1.248 | 7.92E-06 | NM_001198766 |
| Hgf | 0.666 | 2.31E-05 | NM_001289460 |
| Gdf6 | 0.539 | 0.00152 | NM_013526 |
|  |  |  |  |
| Il1r1 | 0.461 | 0.005322 | NM_008362 |
| Thy1 | -0.7298 | 0.000568 | NM_009382 |
